# TDP-43 proteinopathy in Theiler’s murine encephalomyelitis virus infection

**DOI:** 10.1371/journal.ppat.1007574

**Published:** 2019-02-11

**Authors:** Katsuhisa Masaki, Yoshifumi Sonobe, Ghanashyam Ghadge, Peter Pytel, Raymond P. Roos

**Affiliations:** 1 Departments of Neurology, University of Chicago Medical Center, Chicago, IL, United States of America; 2 Departments of Pathology, University of Chicago Medical Center, Chicago, IL, United States of America; University of California, Irvine, UNITED STATES

## Abstract

TDP-43, an RNA-binding protein that is primarily nuclear and important in splicing and RNA metabolism, is mislocalized from the nucleus to the cytoplasm of neural cells in amyotrophic lateral sclerosis (ALS), and contributes to disease. We sought to investigate whether TDP-43 is mislocalized in infections with the acute neuronal GDVII strain and the persistent demyelinating DA strain of Theiler’s virus murine encephalomyelitis virus (TMEV), a member of the *Cardiovirus* genus of *Picornaviridae* because: i) L protein of both strains is known to disrupt nucleocytoplasmic transport, including transport of polypyrimidine tract binding protein, an RNA-binding protein, ii) motor neurons and oligodendrocytes are targeted in both TMEV infection and ALS. TDP-43 phosphorylation, cleavage, and cytoplasmic mislocalization to an aggresome were observed in wild type TMEV-infected cultured cells, with predicted splicing abnormalities. In contrast, cells infected with DA and GDVII strains that have L deletion had rare TDP-43 mislocalization and no aggresome formation. TDP-43 mislocalization was also present in neural cells of TMEV acutely-infected mice. Of note, TDP-43 was mislocalized six weeks after DA infection to the cytoplasm of oligodendrocytes and other glial cells in demyelinating lesions of spinal white matter. A recent study showed that TDP-43 knock down in oligodendrocytes in mice led to demyelination and death of this neural cell [1], suggesting that TMEV infection mislocalization of TDP-43 and other RNA-binding proteins is predicted to disrupt key cellular processes and contribute to the pathogenesis of TMEV-induced diseases. Drugs that inhibit nuclear export may have a role in antiviral therapy.

## Author summary

TDP-43 is a widely expressed nuclear protein that shuttles between the nucleus and cytoplasm, and regulates many aspects of RNA processing, such as splicing, trafficking, stabilization, and miRNA production. In almost all cases of ALS, neuronal and glial TDP-43 is phosphorylated, cleaved, and mislocalized to the cytoplasm, where it aggregates into stress granules and insoluble inclusion bodies. Although the mechanisms involved in TDP-43 proteinopathy remain unclear, impaired nucleocytoplasmic trafficking is thought to play an important role. Here we investigated whether TDP-43 proteinopathy also occurs during TMEV infection since TMEV L protein is known to perturb nucleocytoplasmic transport. We found evidence of TDP-43 proteinopathy in both TMEV-infected cultured cells, with predicted splicing abnormalities, as well as in neural and glial cells of TMEV-infected mice. The findings suggest that TDP-43 may contribute to the pathogenesis of TMEV-induced diseases, including TMEV-induced immune-mediated demyelination.

## Introduction

Trans-activation response (TAR) DNA-binding protein of 43 kDa (TDP-43) is an RNA-binding protein (as well as DNA-binding protein) primarily present in the nucleus and important in RNA processing, mRNA transport/stability, and mRNA translation [2–4]. A variety of cellular stresses normally triggers TDP-43 to transiently shuttle into the cytoplasm and assemble into stress granules (SGs). Due to an abnormality of nucleocytoplasmic transport that is known to occur in amyotrophic lateral sclerosis (ALS), TDP-43 accumulates in insoluble aggregates in the cytoplasm of glia and degenerating neurons in the central nervous system (CNS) [5–7]. The mislocalized TDP-43 is cleaved into C-terminal fragments (CTFs), phosphorylated, and/or ubiquitinated [8–10].The importance of TDP-43 in disease pathogenesis is evidenced by the fact that mutant TDP-43 is a rare cause of familial ALS and, like wild type (wt) TDP-43, is mislocalized to the cytoplasm.

TDP-43 proteinopathy has been described in a number of diseases in addition to ALS [11]. Since the leader (L) protein of Theiler’s murine encephalomyelitis virus (TMEV), a member of the *Cardiovirus* genus of *Picornaviridae*, is known to disrupt nucleocytoplasmic transport [12, 13], we wondered whether TDP-43 proteinopathy occurs in infections with this pathogen; however, it is known that different RNA binding proteins and different protein compositions of the nuclear pore complex are present in different cell types [14]. TMEV includes strains of two subgroups with different disease phenotypes in mice [15]. GDVII strain and other members of the GDVII subgroup do not persist, but cause an acute fatal gray matter disease. In contrast, DA strain and other members of the TO subgroup induce a subclinical acute gray matter disease followed by an immune-mediated demyelinating myelitis with virus persistence in the CNS for the life of the mouse. DA-induced demyelinating disease serves as an experimental model of multiple sclerosis (MS).

Here we report that TMEV infection of cultured cells causes L-dependent mislocalization of TDP-43, and L-independent cleavage and phosphorylation of TDP-43 along with splicing abnormalities. Mislocalization and phosphorylation of TDP-43 also occurs in neuronal cells following early TMEV infection of mice, and in oligodendroglia and other glial cells in demyelinated areas 6 weeks after DA virus infection. These results suggest that TDP-43 mislocalization occurs and presumably contributes to cellular dysfunction and death in TMEV infections. An important role for TDP-43 mislocalization in TMEV-induced demyelinating disease is suggested by recent findings that TDP-43 binds to mRNAs encoding myelin genes, and that a knockdown of TDP-43 in oligodendrocytes of mice leads to demyelination and the death of this neural cell [1].

## Results

### Cytoplasmic mislocalization of TDP-43 induced by TMEV infection

**Fig 1:**
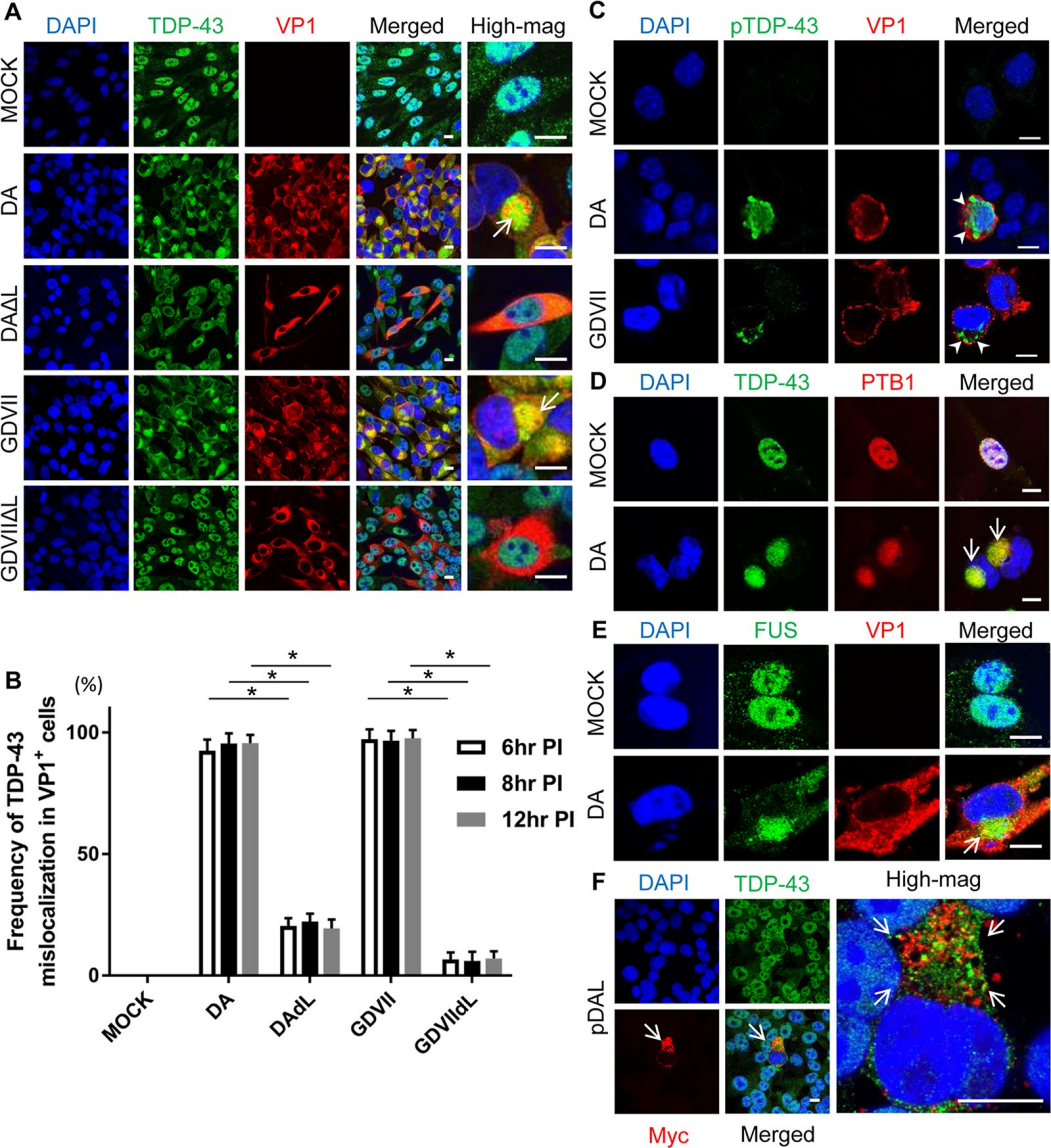
Mislocalization of TDP-43 in TMEV-infected cultured cells. (A, D, E) Immunofluorescent staining of TDP-43, PTB1, and FUS in BHK-21 cells at 8 HPI. (A) TDP-43 is located in the nucleus of mock-infected (VP1-negative) cells. In DA and GDVII infections TDP-43 is depleted from the nucleus and mislocalizes to the cytoplasm of VP1-positive cells where it aggregates (*arrows*). In contrast, DAΔL and GDVIIΔL infections fail to induce TDP-43 mislocalization. (B) Frequency of TDP-43 mislocalization in TMEV-infected cells at different HPI. DA and GDVII infections induce mislocalization of TDP-43 in almost all VP1-positive cells that begins at least as early as 6 HPI and lasts for at least 12 HPI. In contrast, infection with TMEVΔL virus infrequently leads to TDP-43 mislocalization. (C) pTDP-43 is present in the cytoplasm of DA and GDVII-infected L929 cells (*arrowheads*). (D, E) PTB1 (D) and FUS (E) are mislocalized to the cytoplasm in DA infection (*arrows*). (F) 48hs after transfection with pDAL, TDP-43 is mislocalized and aggregates (*arrows*) in the cytoplasm of L-expressing BHK-21 cells (indicated by Myc positivity). Scale bars: 10 μm. *P < 0.001.

In control mock-infected BHK-21 cells, expression of TDP-43 was primarily restricted to the nucleus (Fig 1A). Following infection with DA or GDVII virus, which was detected by positive staining for TMEV VP1 capsid protein, TDP-43 was depleted from the nucleus and aggregated in the cytoplasm (Figs 1A, 1B and S1). The location of TDP-43 was juxtanuclear in structures that resembled aggresomes (see below), which have been previously observed in TMEV-infected cells [16, 17]. In addition, phosphorylated TDP-43 (pTDP-43) was present in the cytoplasm of TMEV-infected cells (Figs 1C and S2).

We questioned whether other RNA-binding proteins were also mislocalized to the cytoplasm in TMEV-infected cells. For this reason, we investigated the localization in cells of i) fused in sarcoma (FUS), which like TDP-43 is a cause of familial ALS when mutated, and ii) polypyrimidine tract binding protein (PTB), which is known to be mislocalized in TMEV infections, where it plays a role in TMEV translation [18, 19]. DA infection induced cytoplasmic mislocalization of both FUS and PTB1, one of PTB isoforms, along with TDP-43 (Fig 1D and 1E).

Since TMEV L protein is known to disrupt nucleocytoplasmic trafficking, we investigated TDP-43 localization following infection with mutant TMEV that had an L deletion. As predicted, DAΔL and GDVIIΔL infection failed to induce mislocalization of TDP-43 in VP1-positive cells (Fig 1A and 1B), demonstrating that TDP-43 mislocalization is indeed L-dependent. In order to further confirm the importance of TMEV L in TDP-43 mislocalization, we transfected eukaryotic expression constructs pDA L and pGDVII L into BHK-21 cells. Although both of these expression constructs caused cytoplasmic mislocalization of TDP-43 in the three cell lines that were tested (Figs 1F and S3), TDP-43 was present in small aggregates in the cytoplasm rather than the aggresome that had been detected in wild type (wt) TMEV-infected cells. The different effect of the TMEV L expression constructs was not a result of a different level of L protein expression when compared to TMEV L protein expression (S4 Fig).

In order to confirm the cytoplasmic mislocalization of TDP-43 in TMEV-infected cells, we separated the nucleus and cytoplasm of cultured cells infected with TMEV (S5 Fig). The results confirmed the prominent TDP-43 mislocalization in infected cells. Some TDP-43 is present in the cytoplasm of mock and TMEVΔL-infected cells presumably due to the normal shuttling of this protein from the nucleus.

### Aggresome formation in TMEV-infected BHK-21 and L929 cells, but not HeLa cells

**Fig 2:**
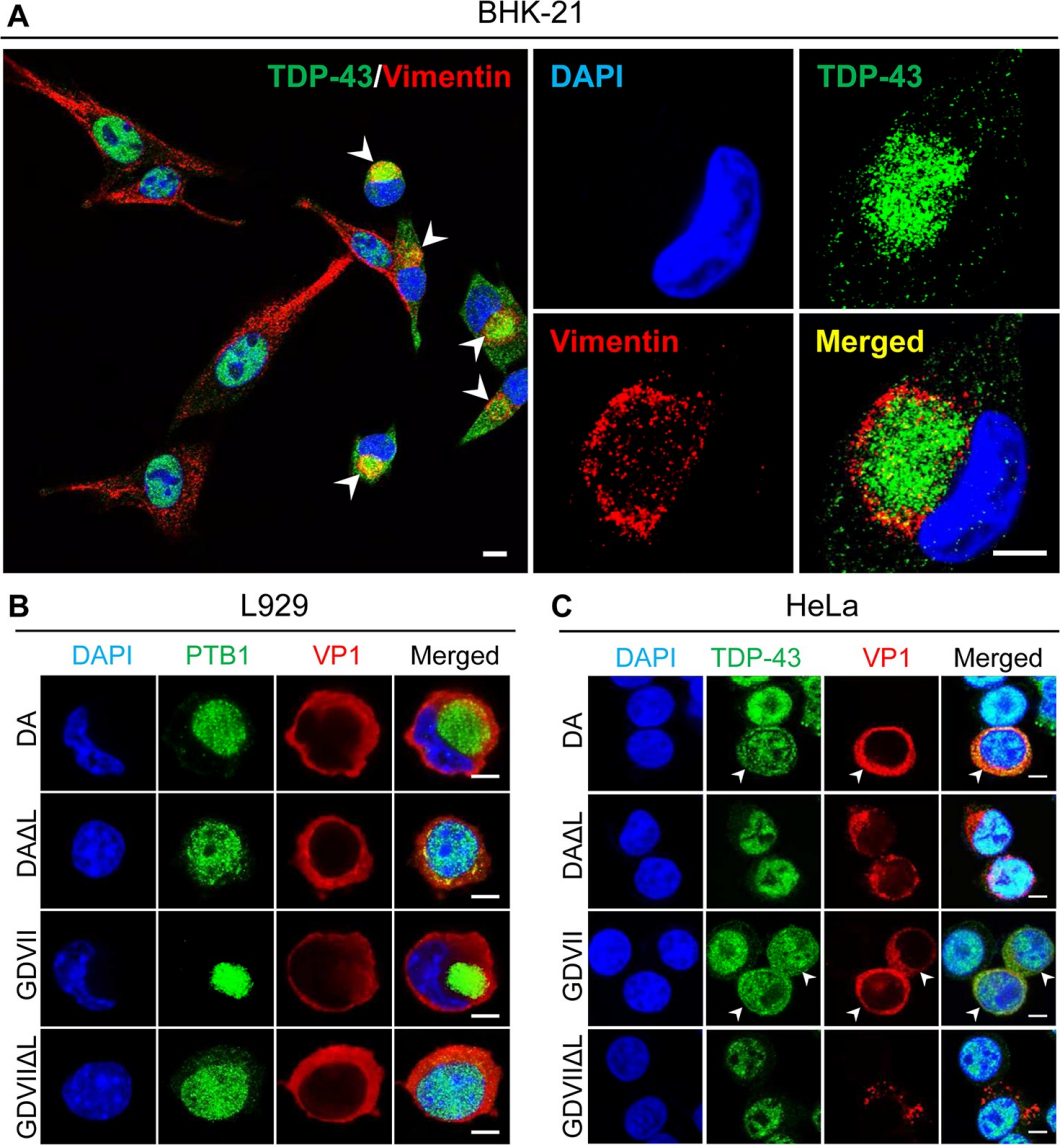
TMEV infection induces aggresome formation in rodent, but not human cells. (A) Double immunofluorescent staining for TDP-43 and vimentin in DA-infected BHK-21 cells at 8 HPI. Cells have a large juxtanuclear structure covered by vimentin that represents an aggresome (*arrowheads*). TDP-43 is localized within the aggresome. (B) DA and GDVII infection in L929 cells leads to cytoplasmic mislocalization and aggregation of PTB1, while DAΔL and GDVIIΔL infections induce minimal mislocalization of PTB1 at this time. (C) HeLa cells infected by DA and GDVII show mislocalization of TDP-43, however, no aggresome is induced (*arrowheads*). DAΔL and GDVIIΔL infection do not induce TDP-43 mislocalization. Scale bars: 10 μm (A) and 5 μm (B, C).

As noted above, the juxtanuclear location of TDP-43 seen following TMEV infection had a morphology typical of an aggresome. Vimentin surrounded these juxtanuclear structures (Fig 2A), as is true in the case of aggresomes [20]. TMEV infections of L929 cells also induced a juxtanuclear aggresome that contained PTB1 (Fig 2B). In contrast, TDP-43 was diffusely present in the nucleus and cytoplasm of DA- and GDVII-infected HeLa cells (Figs 2C and S6), and not in an aggresome, perhaps related to the poor growth of TMEV in these cells [21].

**Fig 3:**
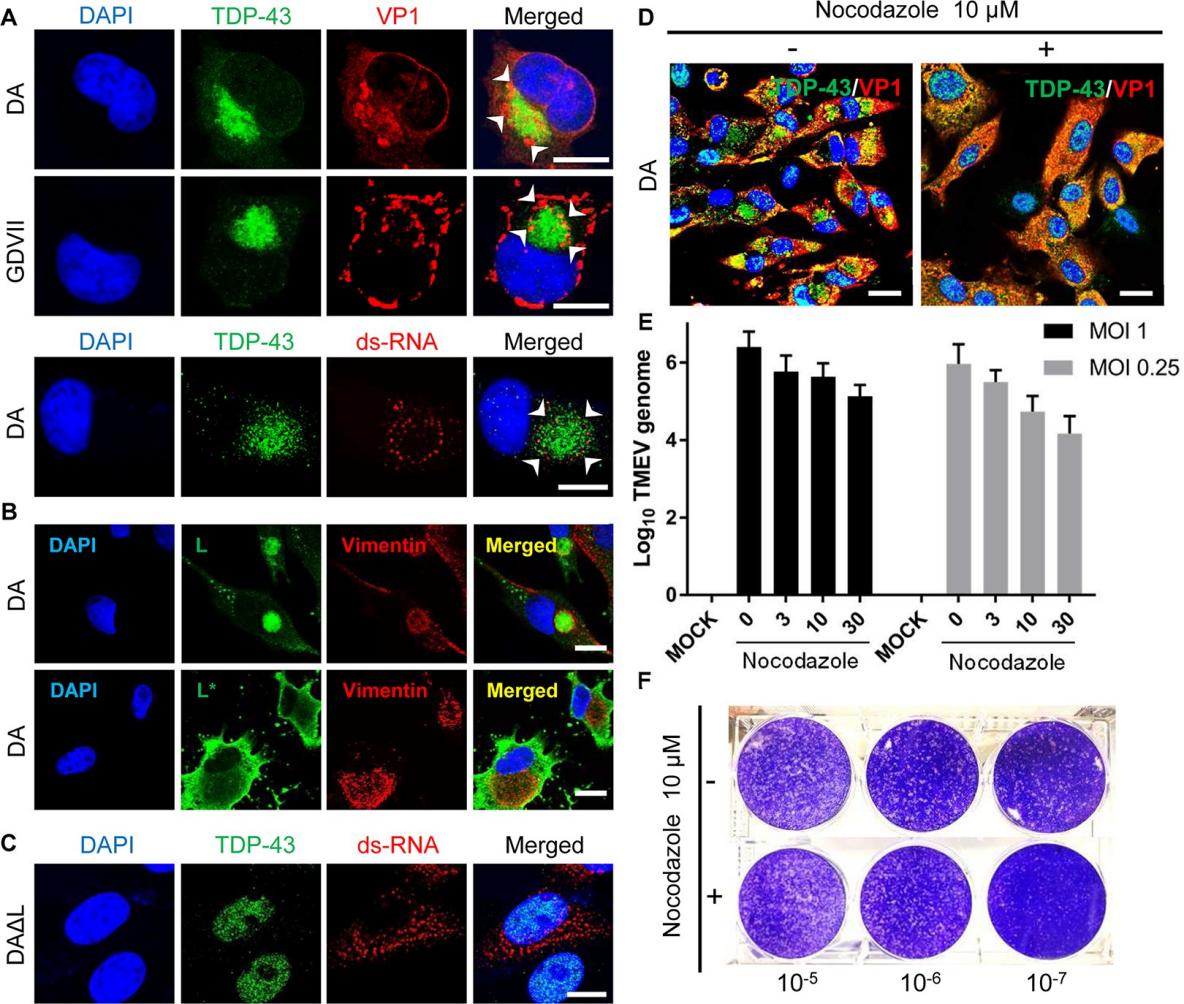
The aggresome is important in TMEV replication. (A, B) DA and GDVII virus-infected BHK-21 cells at 8 HPI. The aggresome in TMEV-infected cells contains VP1, ds-RNA (A) and L (B). L* is expressed in cytoplasm but outside of aggresome (B). (C) ds-RNA is present in small aggregates throughout the cytoplasm of DAΔL-infected BHK-21 cells at 8 HPI. (D) Nocodazole, a microtubule inhibitor, hampers aggresome formation in DA-infected BHK-21 cells at 8 HPI. (E) The amount of DA virus genome is decreased in a dose-dependent manner 6 HPI with two different MOIs in BHK-21 cells that had been treated with nocodazole (0, 3, 10, 30 μM) for 1h prior to infection. (F) Plaque assay of DA-infected BHK-21 cells treated with nocodazole treatment (10 μM, for 1h prior to infection) shows a decrease in virus titer compared to untreated cells. Scale bars: 10 μm.

Aggresomes result from a remodeling of intracellular membranes to generate sites of virus replication [20]. Fig 3A shows that VP1 and double-stranded RNA (ds-RNA), produced during TMEV replication, decorated the margins of aggresomes in TMEV-infected BHK-21 cells; an orthogonal view demonstrates that there is only very partial colocalization of VP1 and TDP-43 (S7 Fig). DA L was present within the aggresome’s vimentin cage, while DA L*, a non-structural protein that inhibits RNase L, was in the cytoplasm, but outside the aggresome (Fig 3B). Although ds-RNA was detected in DAΔL virus-infected BHK-21 cells, it tended to be present in small aggregates throughout the cytoplasm (Fig 3C). VP1 generally had a similar localization to that found with dsRNA in DAΔL virus-infected cells, however, at times it was diffusely distributed in the cytoplasm, presumably related to increasing virion production over time (see later).

In order to assess the importance of aggresomes in TMEV infection, we made use of nocodazole, a microtubule inhibitor that interferes with aggresome formation. BHK-21 cells were exposed to nocodazole (10 μM, 1hr), and then infected with DA virus. Compared to levels obtained with no nocodazole treatment, nocodazole led to a 10-fold reduction in virus genome at an MOI of 1, and 100-fold reduction at an MOI of 0.25 (Fig 3E). As expected, nocodazole treatment decreased the virus titer by more than 10-fold at 12 HPI (Fig 3F). In contrast, the effect of nocodazole on the level of viral genome and infectivity was relatively small in HeLa cell (S8 Fig). These findings suggest that the effect of nocodozole on TMEV replication is not related to this drug’s general disruption of the cytoskeleton, but a more specific effect on aggresomes.

### TMEV infection and stress granules (SGs)

SGs are mainly composed of stalled translation preinitiation complexes, markers such as G3BP1, eIF3A, and TIA1, and RNA-binding proteins including TDP-43. These structures are cytoplasmic non-membranous structures that appear in cells exposed to various stresses, including virus infections [22]. Certain viruses are known to induce SGs while others inhibit SG formation [23]. At times of stress or following treatment with a SG inducer, there is formation of SGs < 1 μm or 1–2 μm in size (S9 Fig).

**Fig 4:**
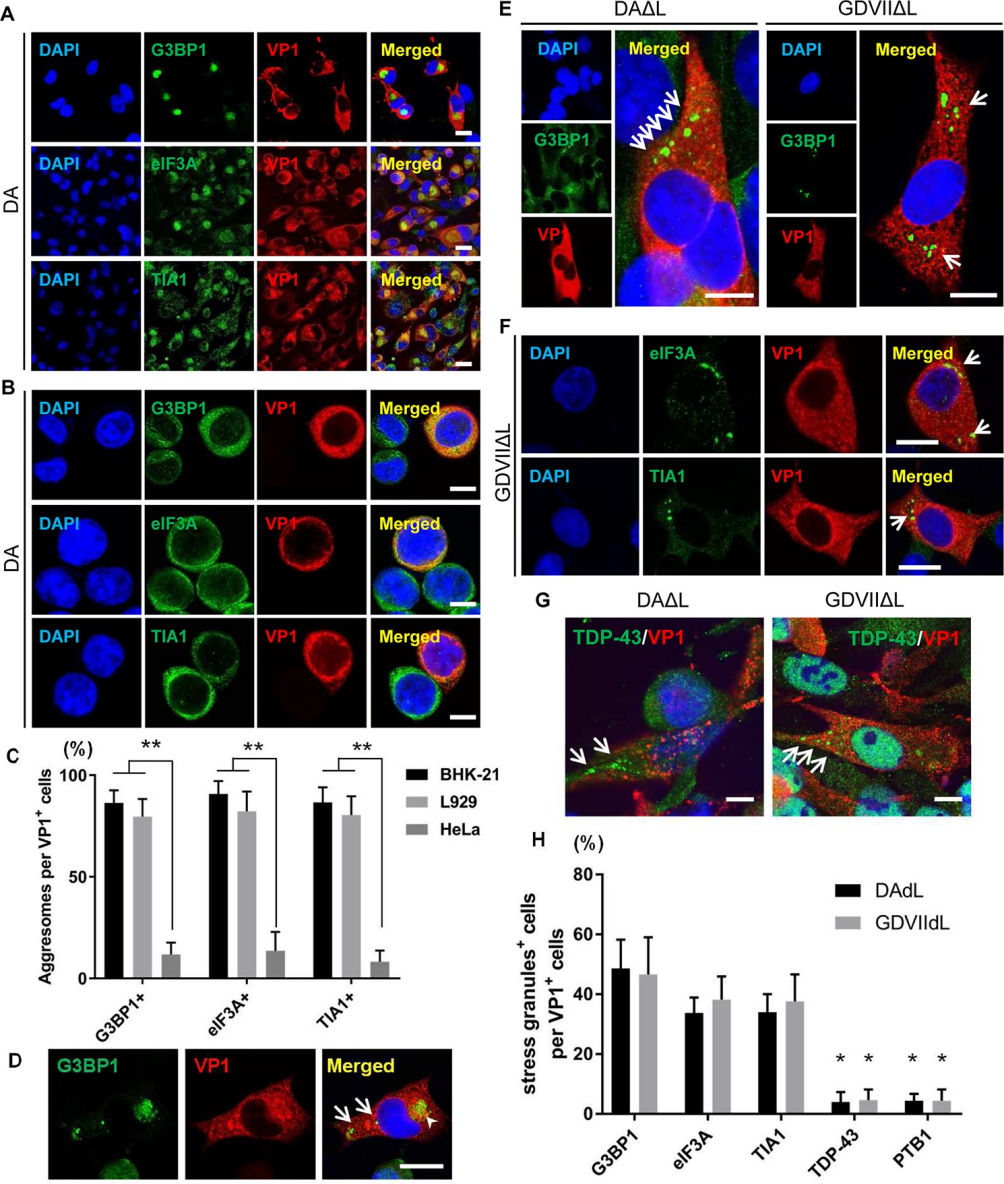
Differences in SG formation in TMEV wt and ΔL infection. (A-C) G3BP1, eIF3A and TIA1 are localized in aggresomes in DA virus-infected BHK-21 (A) and L929, but not HeLa cells (B). (D) G3BP1-positive SGs (*arrows*) and the aggresome (*arrowhead*) are rarely found in the cytoplasm of DAΔL-infected BHK-21 cells at 8 HPI. (E, F) Immunofluorescent staining of BHK-21 cells infected with DAΔL and GDVIIΔL virus at 8 HPI. (E) G3BP1-positive SGs are present in the cytoplasm of VP1-positive cells (*arrows*), while uninfected cells have homogeneous cytoplasmic G3BP1 staining. (F) SGs in GDVIIΔL virus-infected cells contain eIF3A and TIA1 (*arrows*). (G, H) Following infection with DAΔL and GDVIIΔL, TDP-43 and PTB1 are present in structures that resemble SGs (*arrows*); however, they infrequently colocalize with SG markers. Scale bars: 10 μm. *P < 0.01, **P < 0.001.

Borghese and Michiels [24] previously reported that DA L inhibits SG formation in HeLa cells, a human cell line. We examined this issue in HeLa cells as well as two rodent cell lines. Uninfected control BHK-21 cells have homogeneous cytoplasmic immunostaining of SG markers G3BP1, eIF3A and TIA1 (Figs 4 and S9). In DA- and GDVII-infected (rodent) BHK-21 and L929 cells, but not in infected HeLa cells, these markers are located in the aggresome of VP1-expressing cells and not in SGs (Figs 4A–4C and S10); the lack of aggresome formation in TMEV-infected HeLa cells may be associated with the inefficient TMEV infection described in these cells [21]. At times, a VP1-expressing BHK-21 cell expressed these markers in what appeared to be typical SG structures as well as aggresomes, suggesting that the markers (and RNA-binding proteins) may transiently assemble in SGs, and then over time, when there is increasing virus production, relocalize in aggresomes (Fig 4D). Other picornavirus infections are reported to also transiently induce SG formation, followed by an inhibition of SGs later in infection [23]. In the case of TMEVΔL virus-infected cells, typical SGs were induced that immunostained with G3BP1, eIF3A and TIA1 (Figs 4E, 4F and S10), indicating that L interferes with SG formation. The SGs induced by TMEVΔL virus infections rarely colocalized with TDP-43 and PTB1 (Fig 4G and 4H).

### L-independent cleavage of TDP-43 in TMEV-infected BHK-21 cells

**Fig 5:**
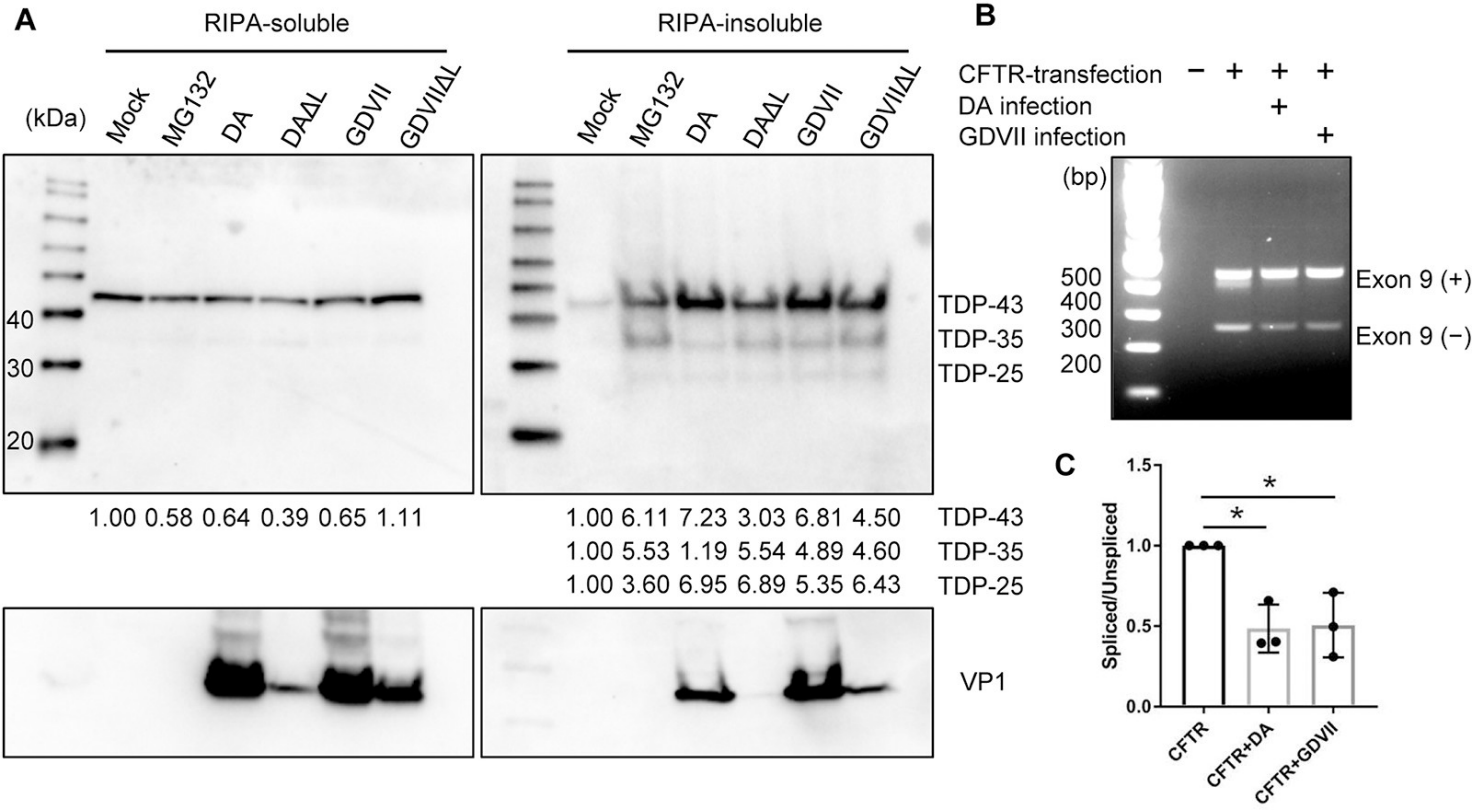
TMEV infection induces cleavage of TDP-43 and abnormal splicing. (A) Western blot of BHK-21 cells that are either uninfected or 8 hours after infection with DA, DAΔL, GDVII or GDVIIΔL virus. As a positive control for cleavage of TDP-43, BHK-21 cells were treated with 10 μM MG-132 for 8hrs. Western blots of cell lysates were immunostained with antibody against TDP-43 (C-terminal) and VP1. In addition to the predicted full-length normal 43-kDa band, 35-kDa and 25-kDa bands are prominently seen in the RIPA-insoluble fraction from wt and TMEVΔL virus-infected cells. The levels of full-length and cleaved TDP-43 were quantitated by densitometric analysis using NIH ImageJ and presented under each blot. The value of each of the bands in the MG132-treated or infected cells was compared to the uncleaved band in mock, which was set to 1. (B) Representative agarose gel electrophoresis of RT-PCR products in CFTR minigene-transfected cells that were or were not infected with DA or GDVII virus. Exon 9 included (+) and excluded (–) RT-PCR products are shown. (C) Spliced versus unspliced ratios were calculated and then normalized to the value of L929 cells that received vector, but were not infected. Mean values are from three different experiments performed. *P < 0.001.

To determine whether TMEV infection induces cleavage of TDP-43, as in the case of ALS, we carried out Western blots on RIPA-soluble and insoluble (but urea soluble) fractions extracted from TMEV-infected BHK-21 cell lysates at 8 HPI. Following infection with both wt and TMEVΔL virus, ~35-kDa and ~25-kDa bands as well as the expected 43-kDa band of full-length TDP-43 were detected in the urea-soluble, but not RIPA-soluble fraction, of BHK-21 cell lysates (Fig 5A). These findings suggest that L-independent cleavage of TDP-43 occurs in BHK-21 cells. Of note, there was no clear correlation between TDP-43 cleavage and TMEV infection, as monitored by VP1 immunodetection.

### Splicing abnormalities in TMEV-infected cells

TDP-43 is known to have an important role in alternative splicing, including cystic fibrosis transmembrane conductance regulator (CFTR) exon 9 skipping [25]. In order to assess splicing abnormalities in infected cells, we transfected L929 cells with a CFTR minigene construct. Compared to uninfected cells, DA or GDVII virus-infected cells had a decrease of the lower band, which corresponds to the exon 9 spliced product (Fig 5B and 5E). These results provide evidence of impaired splicing regulatory activity in the infected cells, presumably because of abnormal TDP-43 localization and aggregation associated with TMEV infection.

### Cytoplasmic mislocalization of TDP-43 in neurons of GDVII virus-inoculated mice

**Fig 6:**
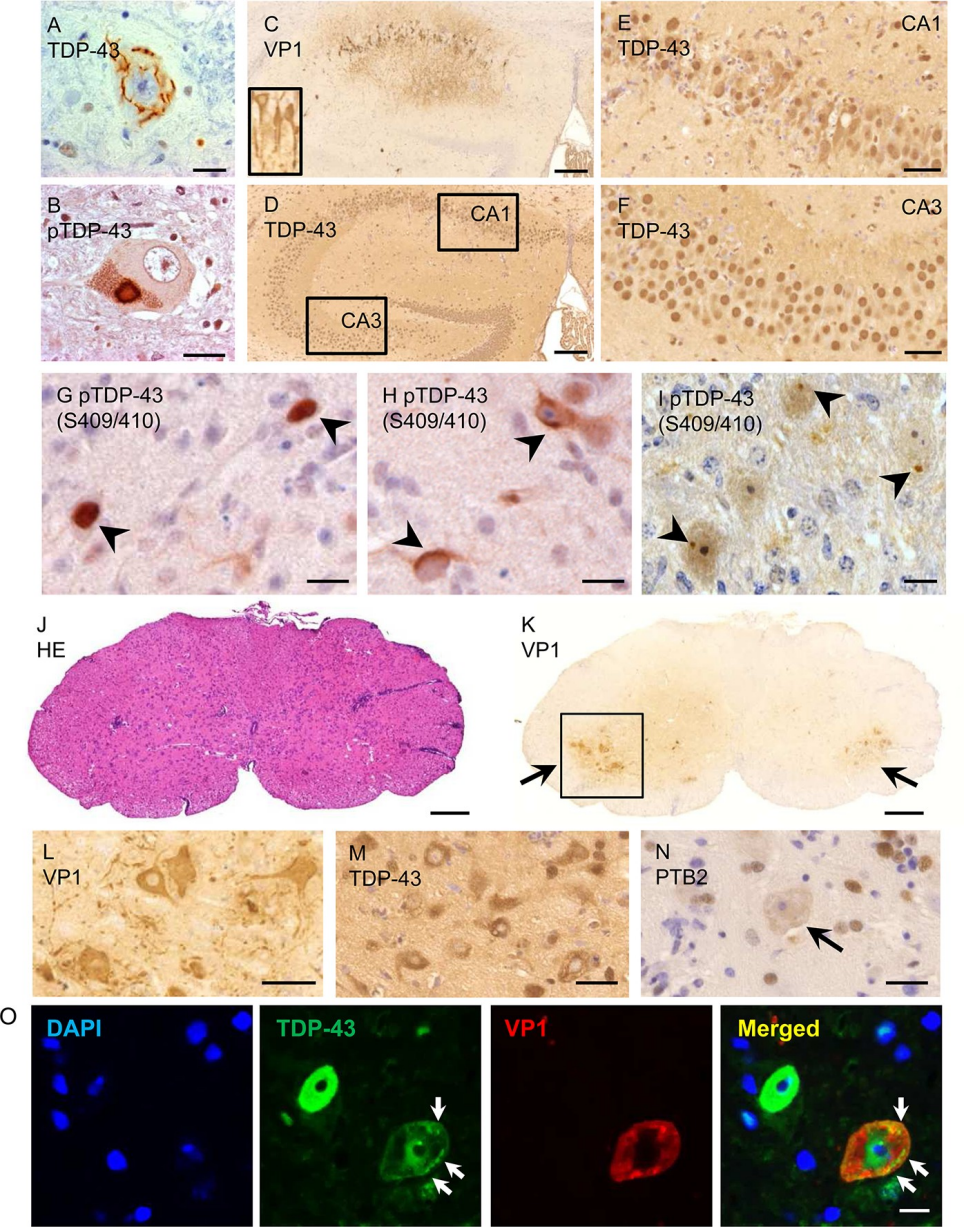
TDP-43 mislocalization and phosphorylation in the CNS 1 week after infection with GDVII virus. (A, B) TDP-43 is depleted from the nucleus of motor neurons in the anterior horn of an ALS patient, and is phosphorylated and mislocalized into the cytoplasm into skein-like inclusion. (C-O) GDVII virus-inoculated mice at 1 week following infection. (C) immunohistochemical staining for VP1 shows GDVII-infected hippocampal CA1 neurons and axons (*inset*). TDP-43 is mislocalized within the cytoplasm of infected hippocampal CA1 neurons (D, E), but not in uninfected CA3 neurons (F). pTDP-43 is present in the nucleus (G) and cytoplasm (H) of infected hippocampal neurons. (I) pTDP-43 is present as a small aggregate in infected brainstem neurons. (J) Haematoxylin and eosin (HE) staining shows perivascular inflammatory infiltrates in the lumbar spinal cord. (K, L) Numerous VP1-positive cells are observed in the anterior horn at two magnifications. (L-O) Higher magnification of the anterior horn shown in I. (L) Immunoreactivity for VP1 is seen in motor neurons and axons. (M) Expression of TDP-43 in the nucleus of anterior horn cells is decreased and mislocalized to the cytoplasm. (N) PTB2 is depleted in the nucleus of anterior horn cells (*arrow*). (O) TDP-43 is present in the nucleus of an uninfected cell, while a VP1-positive motor neuron has decreased TDP-43 staining in the nucleus with cytoplasmic round and linear aggregates (*arrows*). Scale bars: 200 μm (C, D, J, K), 50 μm (E, F), 10 μm (G-I, O) and 20 μm (A, B, L-N).

In the case of ALS and ALS/FTD, TDP-43 is depleted in nuclei of neural cells, and mislocalized and phosphorylated in inclusions in the cytoplasm (Fig 6A and 6B). In order to determine whether the findings that we observed in cultured cells were also present in TMEV-induced disease, we carried out immunohistochemical staining of the CNS of mice 1 week following infection with GDVII virus, a time when mice are paralyzed and near moribund. Neurons in the CA1 region of the hippocampus had VP1 immunostaining (Fig 6C) with mislocalization of TDP-43 to the cytoplasm (Fig 6D and 6E). pTDP-43 was present in the nucleus (Fig 6G) and cytoplasm (Fig 6H), at times in a compact cytoplasmic inclusion body (Fig 6I). Approximately 60% of VP1-positive cells in TMEV-infected mice had evidence of pTDP-43 (S11 Fig). In contrast, TDP-43 maintained its normal nuclear localization in uninfected CA3 region neurons from the same TMEV-infected mouse (Fig 6F) and in normal uninfected mice (S11 Fig). The spinal cord of infected mice showed perivascular mononuclear infiltrates (Fig 6J) with numerous VP1-positive anterior horn cells (Fig 6K and 6L) that had TDP-43 and PTB2 depleted from the nucleus (Fig 6M and 6N). Immunofluorescent staining confirmed the presence and aggregation of TDP-43 in the cytoplasm of VP1-positive motor neurons (Fig 6O).

### Cytoplasmic mislocalization of TDP-43 in neurons and glia of DA-infected mice

**Fig 7:**
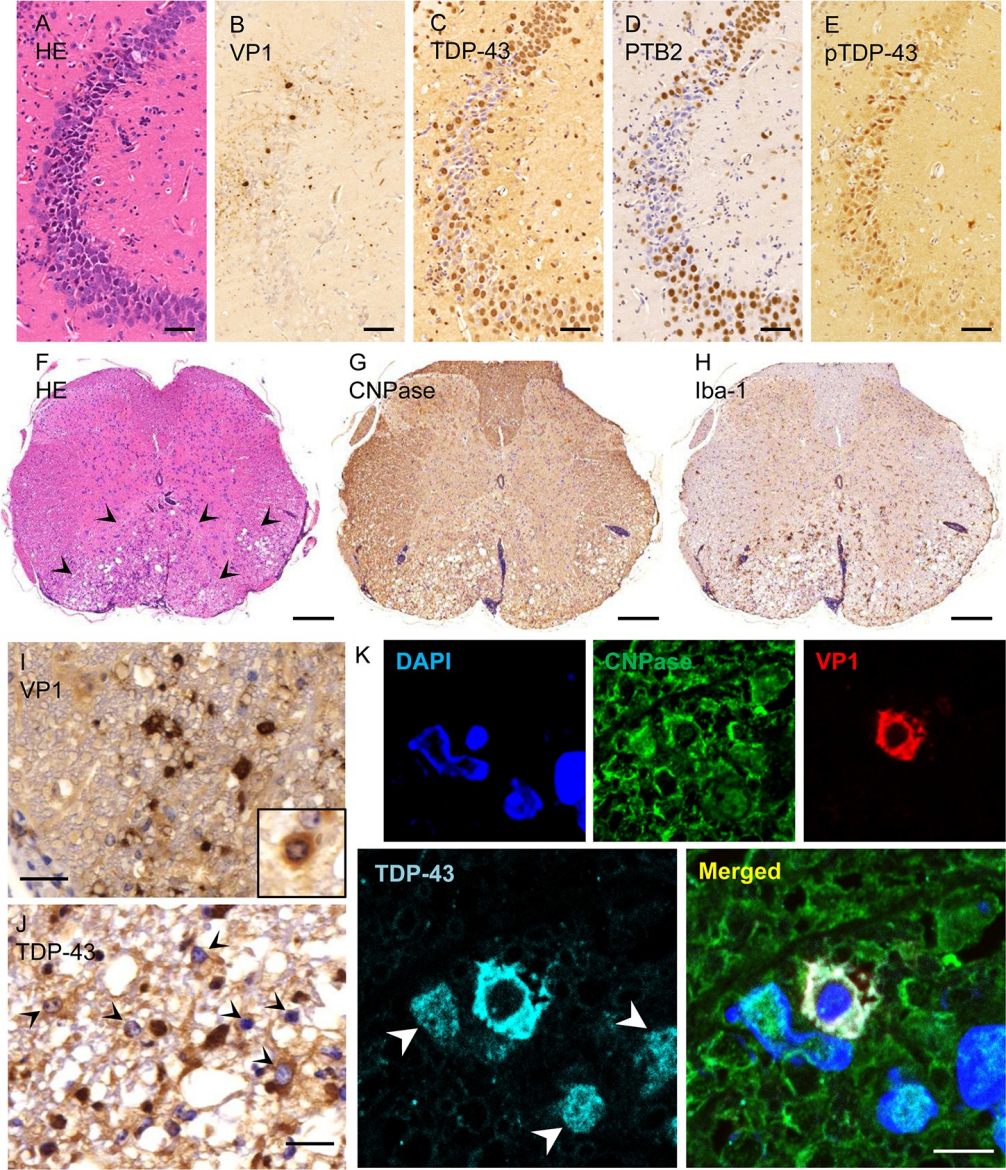
TDP-43 mislocalization and phosphorylation in DA virus-infected mice. (A-E) Serial sections of hippocampus two weeks following infection with DA virus (acute phase). (A-B) Neurons of the CA2 hippocampal region are infected, as indicated by VP1 positivity. (C, D) The expression of TDP-43 and PTB2 is decreased in the nuclei and cells in the infected region. (E) pTDP-43 is present in the nucleus and cytoplasm of infected CA2 neurons. (F-K) 6 weeks following infection with DA virus (chronic phase). (F-H) Serial sections of thoracic spinal cord. (F) HE stain shows perivascular infiltrates and vacuolation in the ventral part of the cord (arrowheads). (G) Immunostaining for CNPase shows demyelination. (H) Immunostaining for Iba-1 shows accumulation of activated microglia and macrophages in the demyelinated region. (I) In the demyelinated region, immunoreactivity for VP1 is present in glial cells and myelin, including the cytoplasm of an oligodendrocyte (*inset*). (J) TDP-43 is mislocalized to the cytoplasm in glial cells in the demyelinated region (*arrowheads*). (K) Immunofluorescent staining for CNPase, VP1 and TDP-43 in the demyelinated region. A VP1-positive oligodendrocyte shows depletion of TDP-43 from the nucleus and mislocalization to the cytoplasm. Note the normal TDP-43 nuclear staining pattern in uninfected cells (*arrowheads*). Scale bars: 50 μm (A-E), 200 μm (F-H), 20 μm (I, J) and 5 μm (K).

DA virus produces a biphasic disease in SJL mice with minimal or subclinical disease within the first two weeks post-infection, followed by progressive paralysis from an inflammatory demyelination that peaks at 6 weeks post-infection. In the acute phase of DA virus infection, VP1-positive neurons and axons were present in the CA2 region of the hippocampus (Fig 7A and 7B); however, the severity of infection and inflammation was mild compared to that seen in GDVII virus-infected mice. Some cells appeared to have cytoplasmic as well as nuclear staining of TDP-43 and PTB2, a splicing isoform of PTB that is increased in neurons compared to other cell types (Fig 7C and 7D). The infected regions generally had a decrease in TDP-43 staining, perhaps partly because many of the infected cells had pTDP-43 (Fig 7E), which was not stained by the anti-TDP-43 antibody that was used.

Six weeks after infection with DA virus, the ventral region of the thoracic spinal cord showed perivascular mononuclear cell infiltrates, (Fig 7F), demyelination, and vacuolation (Fig 7G). Activated microglia clustered within or around the demyelinated areas (Fig 7H). In these demyelinated areas, TDP-43 was depleted from the nucleus and mislocalized to the cytoplasm of VP1-positive white matter glial cells (Fig 7I and 7J), including oligodendrocytes (Fig 7K).

## Discussion

TDP-43 is a ubiquitously expressed RNA-binding protein that predominantly resides in the nucleus, but shuttles across the nuclear membrane in association with mRNAs [26]. A hallmark of almost all cases of ALS is disruption of nucleocytoplasmic trafficking with cytoplasmic mislocalization, aggregation, cleavage, and phosphorylation of TDP-43 in neural cells [5, 7, 9]. TDP-43 mislocalization is thought to lead to abnormalities of splicing and RNA metabolism with subsequent neuronal dysfunction [4, 27, 28]. It is likely that the cytoplasmic mislocalization of other RNA-binding proteins also contributes to the abnormalities of splicing in ALS [29]. In the present study, we demonstrate that TMEV infection leads to cytoplasmic mislocalization of TDP-43 (as well as FUS and PTB) along with cleavage into products similar in size to those found in ALS [7] and TDP-43 phosphorylation. Importantly, TDP-43 mislocalization was also found in neural cells following acute infections of mice, and in oligodendrocytes and other glial cells in demyelinated regions 6 weeks after DA infection.

As is true of many pathogens, picornaviruses disrupt nucleocytoplasmic trafficking during infection, leading to cellular dysfunction as well as the redistribution and hijacking of nuclear proteins into the cytoplasm for use during virus replication [30, 31]. For example, in infections of cultured cells by coxsackievirus B3 (CVB3), a member of the *Enterovirus* genus of *Picornaviridae*, TDP-43 is mislocalized (by viral protease 2A) and cleaved (by viral protease 3C) [32]. In CVB3 infections, TDP-43 colocalized with SGs in the cytoplasm at 3 HPI, the longest time observed. Human immunodeficiency virus-positive neural cells have also been reported to have TDP-43 in cytoplasmic inclusions [33].

In ALS, TDP-43 is thought to shuttle into the cytoplasm initially into SGs, and then remain aggregated in the cytoplasm. In the case of TMEV-infected BHK-21 and L929 cells, we detected the normal markers for SGs (G3BP1, TIA1 and eIF3A) in aggresomes rather than SGs. Of note, SGs were present following TMEVΔL infections, suggesting that L inhibits SG formation, as has been reported by others [24]. The aggresomes in TMEV-infected cells also contained TDP-43, FUS, PTB1, TMEV proteins (VP1, L), and dsRNA. Nocodazole, a microtubule inhibitor that interferes with aggresome formation, decreased viral replication, suggesting that TMEV uses the aggresome as a “viral factory,” perhaps by concentrating proteins and genome in one region of the cell, as described for other virus infections [34]; however, nocodazole’s disruption of the cytoskeleton with a resultant disturbance of cell physiology may also have had a substantial indirect effect on viral replication. In contrast, TMEV infection of HeLa cells led to minimal cytoplasmic translocation of TDP-43 with no aggresome formation, perhaps a reflection of the reported inefficient infection of these cells [21]; the reasons for the lack of aggresome formation and inefficient infection remain unclear. Cytoplasmic TDP-43 aggregates in ALS have also been referred to as aggresomes [35–37]. In the latter case, the aggresome is thought to be a cytoprotective response that sequesters potentially toxic misfolded proteins and facilitates their clearance by autophagy [20, 38].

Mislocalization and phosphorylation of TDP-43 occurred in TMEV-infected cultured cells as well as neuronal and glial cells of TMEV-infected mice. In DA virus-induced demyelinated regions, TDP-43 and other RNA-binding proteins were mislocalized in glial cells, including oligodendrocytes. The fact that TDP-43 was not present in the cytoplasm following infection with TMEVΔL virus, suggests that L interfered with nucleocytoplasmic transport. TDP-43 mislocalization in neural cells may also be influenced by inflammatory stimuli since tumor necrosis factor-α can lead to mislocalization of TDP-43 [39]. In addition, interferon-γ leads to hnRNP A1 mislocalization and accumulation into the cytoplasm [40].

The mislocalization of RNA-binding proteins in TMEV infections may disrupt cellular splicing and mRNA translation, thereby contributing to neuronal dysfunction and death in GDVII and DA early disease as well as oligodendrocyte dysfunction in the late demyelinating disease of DA-infected mice. Our previous study suggested the possibility that PTB mislocalization in TMEV-infected neurons played a role in neuronal dysfunction [41]. The deleterious effect of PTB2 mislocalization in neurons may be compounded by the FUS mislocalization that was also present, since the latter RNA binding protein is important in axonal transport [42]. Importantly, recent studies have demonstrated that: i) TDP-43 binds to mRNAs of myelin proteins, ii) knockdown of TDP-43 in oligodendrocytes of mice leads to demyelination and death of this neural cell [1]. TMEV L-dependent nucleocytoplasmic trafficking defect is likely to also interfere with other RNA binding proteins in addition to the three that were investigated as well as to disrupt the proper subcellular localization of a number of key transcription factors and proteins in oligodendrocytes and oligodendrocyte precursor cells that are needed for efficient myelination and remyelination [43–46]. Altered nucleocytoplasmic transport leading to mislocalization of RNA-binding proteins and other macromolecules with associated cellular dysfunction may underlie a number of disease states, both infectious as well as non-infectious. The importance of this mechanism of cell dysfunction highlights the potential relevance of antiviral drugs that target nucleocytoplasmic transport.

## Materials and methods

### Ethics statement

The study involving the analysis of human subjects was approved by The University of Chicago Institutional Review Board for Clinical Research. Informed written consent for an autopsy was obtained from an immediate member of the deceased’s family. Animal use was approved by The University of Chicago Institutional Animal Care and Use Committee (IACUC) under the Protocol Number 71772. Animal work conducted at the University of Chicago complies with all applicable provisions of the Animal Welfare Act (AWA) and the Public Health Service (PHS) Policy on Humane Care and Use of Laboratory Animals. The PHS Policy incorporates the standards in the Guide for the Care and Use of Laboratory Animals and the U.S. Government Principles for the Utilization and Care of Vertebrate Animals Used in Testing, Research and Training and requires euthanasia be conducted according to the AVMA Guidelines for the Euthanasia of Animals. The University of Chicago Animal Care Program has an approved Assurance with the National Institute of Health (NIH), is registered with the United States Department of Agriculture (USDA) and is accredited by the Association for Assessment and Accreditation of Laboratory Animal Care International (AAALAC).

### Viruses

DA and GDVII viruses were derived from a full-length infectious cDNA clone [15]. DAΔL virus has a deletion of amino acids 2 to 67 of L [47]. GDVIIΔL virus (originally referred to as dl-L virus) [48] has a deletion of amino acids 2 to 71 of L, and was previously received as a gift from M. K. Rundell.

### Cultured cells and infections

Infections were carried out in BHK-21 (ATCC, CCL-10), L929 (ATCC, CRL-6364) or HeLa cells (ATCC, CCL-2), usually with a multiplicity of infection (MOI) of 10. BHK-21 cells were used for plaque assays and the growth of virus stocks, as previously described [49].

For the study of TDP-43 cleavage, cells were treated with 1μM of the proteasome inhibitor MG-132 (Cell Signaling Technology, Danvers, MA) for 16hs prior to harvest. For induction of SGs, BHK-21 cells were treated for 45 min with 0.5mM sodium arsenite (Sigma Aldrich, St Louis, MO). In investigations of the aggresome, nocodazole (Sigma Aldrich), a microtubule inhibitor, was solubilized in DMSO and added at varying concentrations to the culture medium for 1h prior to infection.

### Plasmids and transfection

pDAL and pGDVIIL, which are eukaryotic expression constructs of DA L and GDVII L respectively, with myc/His epitope tags at the carboxyl terminus [47], were transfected into BHK-21 cells using Lipofectamine 3000 (Thermo Fisher Scientific, Waltham, MA).

### Immunocytochemistry and confocal laser microscopy of cultured cells

Cells on coverslips were harvested 8hs post infection (HPI) or 48hs after transfection, fixed in 4% paraformaldehyde for 5 min, and then permeabilized with phosphate buffered saline (PBS) with 0.1% Triton X-100 for 20 min at room temperature. The coverslips were then incubated overnight at 4°C with primary antibodies (S1 Table). After rinsing, cells were incubated for 30 min with Alexa 594-conjugated goat anti-mouse IgG and Alexa 488-conjugated goat anti-rabbit IgG (Invitrogen, Carlsbad, CA), and then counterstained with 4',6-diamidino-2-phenylindole (DAPI). Images were captured using a confocal laser microscope system (Leica TCS SP5, Leica Microsystems, Wetzlar, Germany). A sequential multiple fluorescence scanning mode was used to avoid nonspecific overlap of signals. In some experiments, manual counting of infected cells was carried out in five different regions of the coverslips.

### Western blotting

Cells were lysed 8 HPI with a radioimmunoprecipitation assay (RIPA) buffer containing a protease inhibitor and phosphatase inhibitor cocktail (Thermo Fisher Scientific, Waltham, MA). Lysates were centrifuged at 14,000 rpm for 30 min at 4°C, and supernatants collected as RIPA buffer-soluble proteins. The pellets were sonicated and centrifuged twice at 14,000 rpm for 30 min at 4°C to obtain RIPA buffer-insoluble pellets. Pellets were dissolved in urea buffer (8 M urea, 50 mM Tris-HCl, pH 8.5) and then sonicated again prior to electrophoresis. Ten μg of total protein quantified by a Pierce BCA Protein Assay Kit (Thermo Fisher Scientific, Waltham, MA) was subjected to electrophoresis on 10% SDS polyacrylamide gels, and then transferred to Amersham Hybond P 0.45 μm PVDF membrane (GE Healthcare, Buckinghamshire, UK). The membrane was first blocked with 5% non-fat skim milk in Tris-buffered saline (TBS) containing 0.05% Tween-20 for 30 min at room temperature, and then incubated for 1h at room temperature with a rabbit antibody directed against C-terminal TDP-43 (1:1000, Proteintech, Rosemont, IL) or a mouse monoclonal antibody against TMEV VP1 (1:2000), which was previously called GDVII mAb2 [50], or a mouse monoclonal antibody against Lamin A/C (1:1000, Cell Signaling Technology, Danvers, MA), or a mouse monoclonal antibody against β–actin (1:5000, Sigma Aldrich, St Louis, MO). Following washing, the membrane was incubated with anti-rabbit or anti-mouse horseradish peroxidase–conjugated secondary antibodies (GE Healthcare, Buckinghamshire, UK) for 1h at room temperature. The signal was detected using SuperSignal West Dura Extended Duration Substrate (Thermo Fisher Scientific, Waltham, MA), and analyzed using ChemiDoc MP Imaging System (Bio-Rad Laboratories, Hercules, CA).

### Quantitative RT-PCR

DA RNA was extracted from BHK-21 and HeLa cell homogenates using an RNeasy Plus minikit (Qiagen). A region between nt 1485 and 1684 was amplified using forward primer TACTATGGCACCTCTCCTCTTGGA and reverse primer CAGCCGCAAGAACTTTATCCGTTG with a Superscript III Platinum two-step qRT-PCR kit with SYBR green (Invitrogen). A region between nt 182 and 721 of the murine β-actin gene, which was used for normalization and determination of the quality of total mRNA, was amplified using forward primer GTGGGCCGCTCTAGGCACCAA and reverse primer CTCTTTGATGTCACGCACGATTTC. qRT-PCR was conducted on a CFX96 Real-Time System (Bio-Rad). The ΔΔCT method of relative quantitation was used to calculate fold change of DA with β-actin.

### Splicing analysis

In order to assess splicing in TMEV-infected cells, a cystic fibrosis transmembrane conductance regulator (CFTR) minigene construct designed to evaluate CFTR exon 9 splicing (a gift from Virginia Lee’s lab and described in Buratti et al.[25]) was transfected into L929 cells using Lipofectamine LTX reagent (Invitrogen). Twenty-four hours later, the cells were infected separately with DA or GDVII viruses at an MOI of 10. Total RNA was prepared from cells 12 h after infection of viruses, and RT-PCR was performed with 1 μg of total RNA and 1 μl of resulting cDNA. The relative exclusion of exon 9 was evaluated by primer extension from the flanking sequence of exon 9 using the following primers, as previously described [25]: TAGGATCCGGTCACCAGGAAGTTGGTTAAATCA; CAACTTCAAGCTCCTAAGCCACTGC. The PCR products were visualized on a 2% agarose gel. Relative amounts of different splice products were quantified and visualized using Image J. The experiments were repeated in triplicate.

### Human subjects

Immunohistochemical studies were performed on autopsied brain specimens of a patient with ALS.

### Animal studies

TMEV was inoculated intracerebrally in weanling SJL mice (Jackson Laboratory, Bar Harbor, ME), and mice were sacrificed at 1, 2 or 6 weeks post infection (PI). At the time of sacrifice, mice were deeply anesthetized and perfused transcardially first with PBS, and then with 4% paraformaldehyde in 0.1 M phosphate buffer. CNS tissues were fixed in 10% buffered formalin and processed into paraffin sections (5 μm thick). Deparaffinized sections were hydrated in ethanol and then incubated with 0.3% hydrogen peroxide in absolute methanol for 30 min at room temperature to inhibit endogenous peroxidase. After rinsing with tap water, sections were washed twice using Tris–HCl with 0.1% Triton X-100 for 5 min, and then with Tris–HCl for 5 min. Sections were then incubated at 4°C overnight with primary antibody (S2 Table) diluted in 5% normal goat serum, 50 mM Tris-HCl (pH 7.6) and 1% BSA. After rinsing, sections were subjected to labeling by an enhanced indirect immunoperoxidase method. The reaction product was developed using a solution of 3, 3’-diaminobenzidine (DAB). Sections were counterstained with hematoxylin. Double immunostaining was carried out with two enzyme systems, peroxidase and alkaline phosphatase, followed by staining with Vector Red (Vector Laboratories).

Paraffin sections were also used for immunofluorescent staining. Sections were deparaffinized in xylene, rehydrated through an ethanol gradient, and then incubated with primary antibody for 1h at room temperature. The following antibodies were used: mouse monoclonal anti-VP1, rabbit anti-TDP-43, and rabbit anti-2',3'-cyclic nucleotide-3'-phosphodiesterase (CNPase) (S2 Table); rabbit anti-TDP-43 was pre-conjugated with Zenon Alexa Fluor 647 rabbit IgG (Invitrogen). After rinsing, sections were incubated for double immunofluorescence with Alexa 488-conjugated goat anti-rabbit IgG and Alexa 594-conjugated goat anti-mouse IgG (Invitrogen) and for triple immunofluorescent staining with Alexa 488-conjugated goat anti-rabbit IgG, Alexa 555-conjugated goat anti-mouse IgG (Invitrogen), and DAPI. Images were captured using a confocal laser microscope system (Leica TCS SP5, Leica Microsystems, Wetzlar, Germany) with sequential multiple fluorescence scanning mode to avoid non-specific overlap of colors. All photographs were captured under the same magnification, laser intensity, gain and offset values, and pinhole setting.

### Statistical analysis

Statistical analysis was performed by an unpaired t-test or one-way ANOVA with Tukey's multiple comparisons test using GraphPad Prism version 7.0a. A *P*-value of <0.05 was considered significant. The data are presented as the mean ± standard deviation (S.D.).

## Supporting information

S1 Fig Mislocalization of TDP-43 in TMEV infection at two time points. Immunofluorescent staining for TDP-43 in BHK-21 cells at 6 and 12 HPI. TDP-43 cytoplasmic mislocalization and aggregate formation induced by DA and GDVII infection are present by 6 HPI and persists for at least 12 HPI. Scale bars: 10 μm.
(TIF)

S2 Fig DA and GDVII cause phosphorylation of TDP-43 in BHK-21 cells. Double immunofluorescent staining for pTDP-43 and VP1 in BHK-21 cells at 8 HPI. pTDP-43 is present in the cytoplasm of VP1-positive cells infected with DA or GDVII virus. Scale bars: 10 μm.
(TIF)

S3 Fig DAL and GDVIIL cause mislocalization of TDP-43. TDP-43 mislocalization and aggregate formation (*arrowheads*) is present in pDAL- or pGDVIIL-transfected BHK-21, L929, and HeLa cells, The expression of L is indicated by Myc positivity. Scale bars: 10 μm.
(TIF)

S4 Fig L protein expression in pDAL- or pGDVIIL-transfected cells is similar to that seen in DA-infected cells. (A) Representative images of immunofluorescence using anti-L antibody. L is expressed in pDAL- or pGDVIIL-transfected BHK-21 cells (that are detected by Myc staining) and DA-infected BHK-21 cells (that are detected by VP1 staining). (B) Intensity of immunofluorescence for L. The Intensity of immunofluorescence of L within each cell (shown, for example, surrounded by a dotted line in (A)) was measured by ImageJ in 20 cells in 5 random fields and was then plotted as a dot graph. The intensity of immunofluorescence in cells from the three groups is not statistically significant. Scale bars: 10 μm.
(TIF)

S5 Fig Cytoplasmic mislocalization of TDP-43 in DA- or GDVII-infected cells. Western blotting of nuclear (N) and cytoplasmic (C) fractions of TMEV-infected BHK-21 cells. The nuclear and cytoplasmic fractions of BHK-21 cells were separated by using NE-PER Nuclear and Cytoplasmic Extraction Reagents (Thermo Fisher Scientific, Waltham, MA). In MOCK, DAΔL and GDVIIΔL-infected cells, TDP-43 is predominantly expressed in the nucleus (and transiently may enter the cytoplasm). In contrast, TDP-43 is significantly mislocalized to the cytoplasm of DA- and GDVII-infected cells. The expression of LaminA/C, a nuclear envelope protein, is primarily in the nuclear fraction, while TMEV VP1 is present in the cytoplasmic fraction.
(TIF)

S6 Fig DA infection does not cause aggresome formation in HeLa cells. HeLa cells infected by DA virus at 12 HPI. Although TDP-43 is slightly mislocalized to the cytoplasm in VP1-positive cells, aggresomes are not observed in this cell (*arrow*). Scale bar: 5 μm.
(TIF)

S7 Fig Orthogonal view of TDP-43 and VP1 expression pattern in TMEV-infected cells. BHK-21 cells infected by DA and GDVII virus at 8 HPI. Both TDP-43 and VP1 accumulate in the juxtanuclear aggresome. TDP-43 and VP1 are partly co-localized within the aggresome shown in yellow. Scale bars: 5 μm.
(TIF)

S8 Fig Minimal effect of nocodazole treatment on the viral genome and infectivity in DA-infected HeLa cells. (A) The amount of DA virus genome at 6 HPI with two different MOIs in HeLa cells that had been treated with nocodazole (0, 3, 10, 30 μM) for 1h prior to infection. The viral genome is only slightly decreased with the 30 μM nocodazole treatment. (B) Plaque assay of DA-infected BHK-21 cells treated with nocodazole treatment (10 μM, for 1h prior to infection) shows a similar virus titer compared to untreated cells.
(TIF)

S9 Fig Sodium arsenite-induced SGs contain TDP-43 and PTB1. (A-C) BHK-21 cells treated for 45 minutes with 0.5 mM sodium arsenite, a SG inducer, develop SGs that contain SG markers: G3BP1 (A), eIF3A and TIA1 (B). SG markers in mock-treated cells have homogeneous cytoplasmic immunostaining. Following sodium arsenite treatment, SG markers are present in small structures of the typical size of SGs. (C) Following sodium arsenite treatment, TDP-43 and PTB1 partly move into the cytoplasm and merge with TIA1 in SGs (*arrowheads*). Scale bars: 10 μm.
(TIF)

S10 Fig DAΔL virus induces SGs in L929 and HeLa cells. L929 (A) and HeLa (B) cells infected by DAΔL virus at 8 HPI. SGs containing G3BP1, eIF3A and TIA1 are present in the cytoplasm of VP1-positive cells (*arrows*). (C) L929 cells infected by DA virus at 8 HPI. Aggresomes containing G3BP1, eIF3A and TIA1 are observed in VP1-positive cells. Scale bars: 10 μm
(TIF)

S11 Fig The frequency of TDP-43 mislocalization in VP1-positive cells in mice. (A, B) Double immunofluorescence for TDP-43 and VP1 in the hippocampus of uninfected and TMEV-infected mice. (A) TDP-43 is predominantly localized to the nucleus of CA1 region neurons in uninfected mice. In contrast, TDP-43 is depleted in the nucleus, and mislocalized to the cytoplasm of VP1-positive neurons in CA1 region 1 week after infection with GDVII virus. The frequency of TDP-43 mislocalization in VP1-positive cells is ~80%, as shown in the graph bar (n = 3). (B) TDP-43 is depleted in the nucleus and mislocalized to the cytoplasm in VP1-positive neurons (*arrows*) in CA2 region two weeks after infection of DA virus. The frequency of TDP-43 mislocalization in VP1-positive cells is ~70%, as shown in the graph bar (n = 3). (C) Representative image showing phosphorylation of TDP-43 in VP1-positive CA1 region neuron (*arrow*) two weeks after infection of DA virus. Higher magnification shows skein-like inclusion (*arrows*) which is immunopositive for pTDP-43 (*brown*) in a VP1-positive cell (*pink*). The frequency of TDP-43 phosphorylation in VP1-positive cells is ~60%, as shown in the graph bar (n = 3). Scale bars: 10 μm. *P < 0.0001.
(TIF)

S1 Table Antibodies used for immunocytochemistry. (DOCX)

S2 Table Antibodies used for immunohistochemistry. (DOCX)
