# Supplementary figures and images for: TDP-43 proteinopathy in Theiler’s murine encephalomyelitis virus infection
Source: PLoS Pathog. 2019 Feb 11;15(2):e1007574. doi: 10.1371/journal.ppat.1007574 (PMC6390522; doi:10.1371/journal.ppat.1007574)

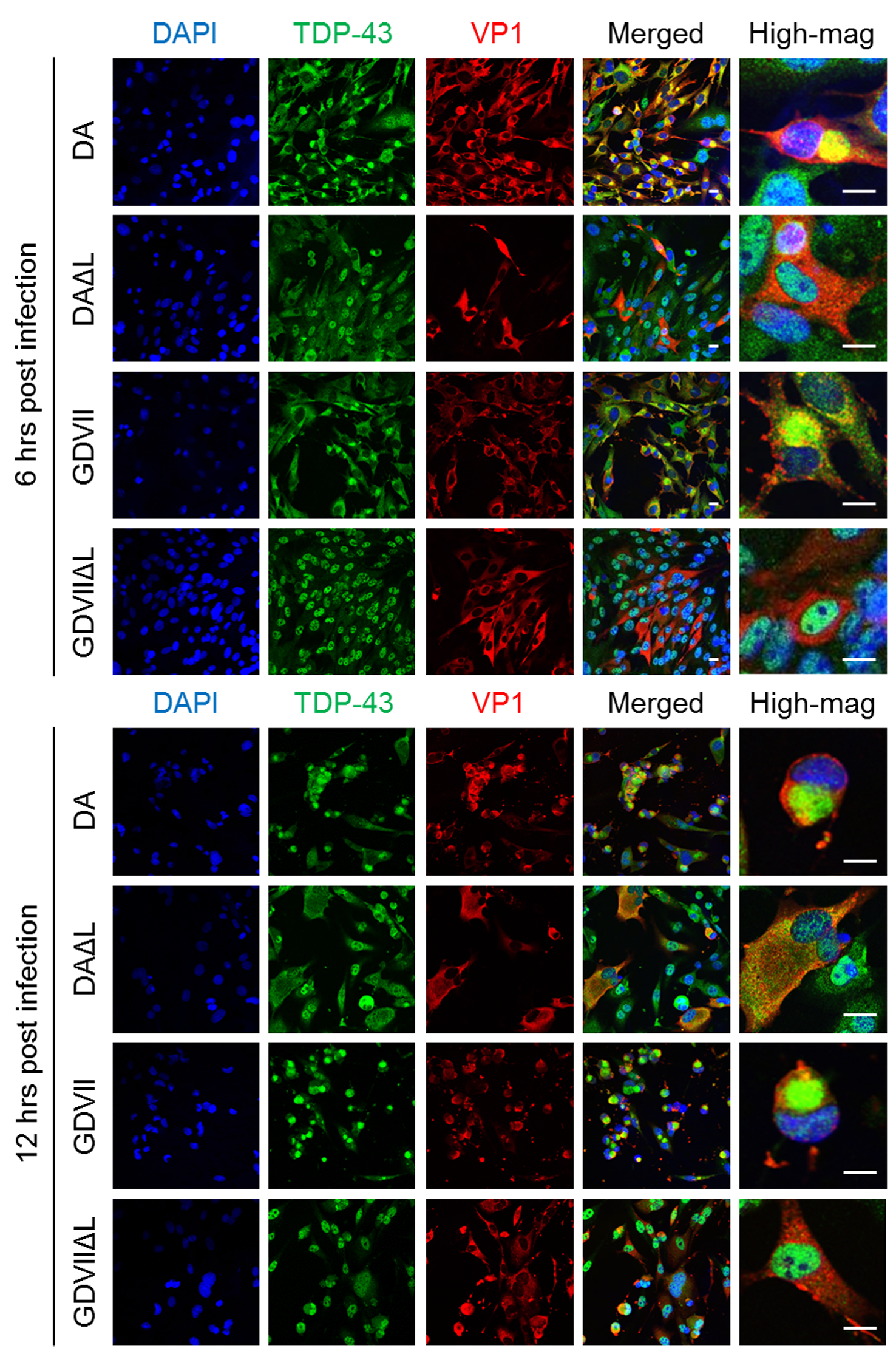

Supplement: S1 Fig — Immunofluorescent staining for TDP-43 in BHK-21 cells at 6 and 12 HPI. TDP-43 cytoplasmic mislocalization and aggregate formation induced by DA and GDVII infection are present by 6 HPI and persists for at least 12 HPI. Scale bars: 10 μm. (TIF) [file ppat.1007574.s001.tif]

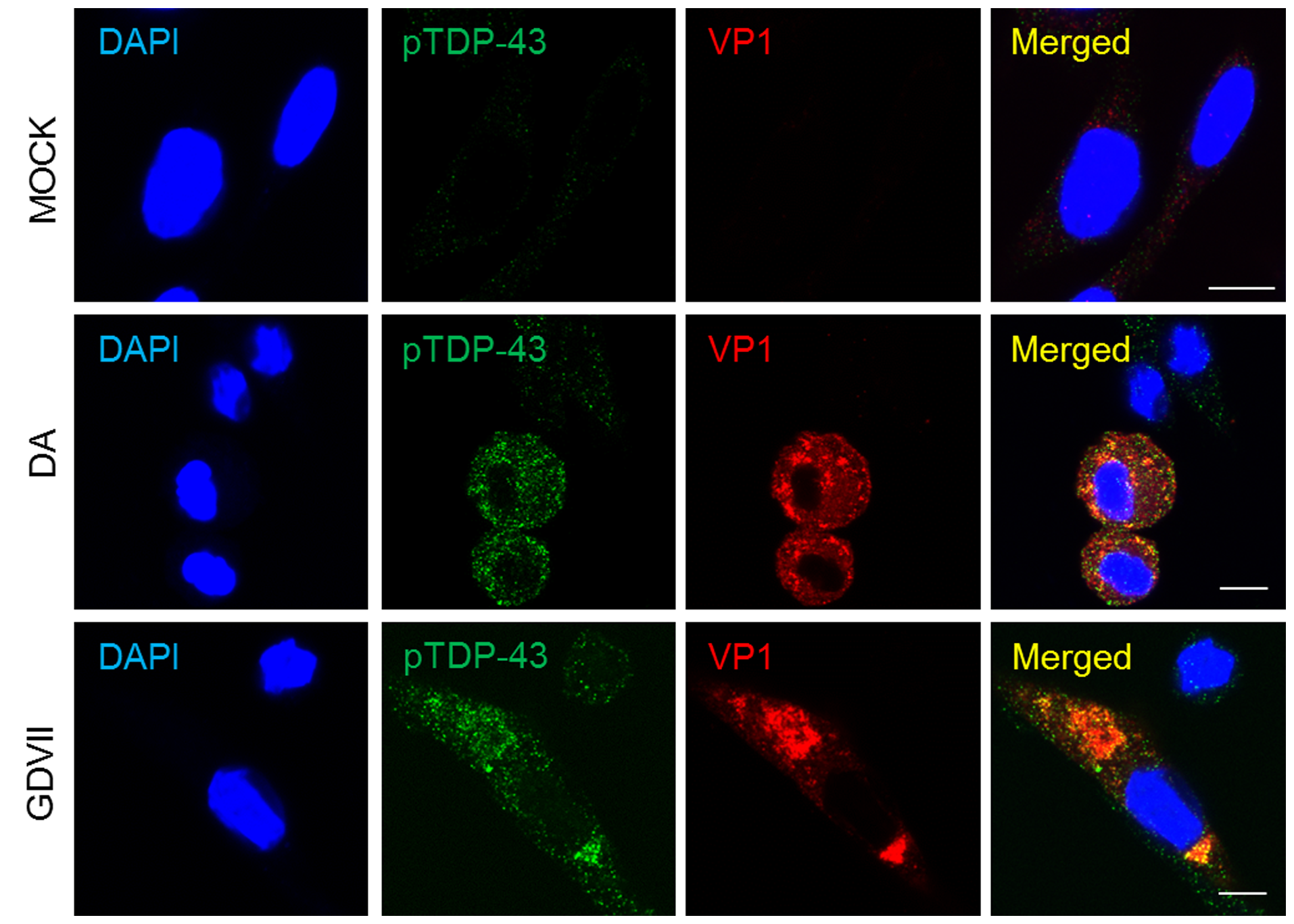

Supplement: S2 Fig — Double immunofluorescent staining for pTDP-43 and VP1 in BHK-21 cells at 8 HPI. pTDP-43 is present in the cytoplasm of VP1-positive cells infected with DA or GDVII virus. Scale bars: 10 μm. (TIF) [file ppat.1007574.s002.tif]

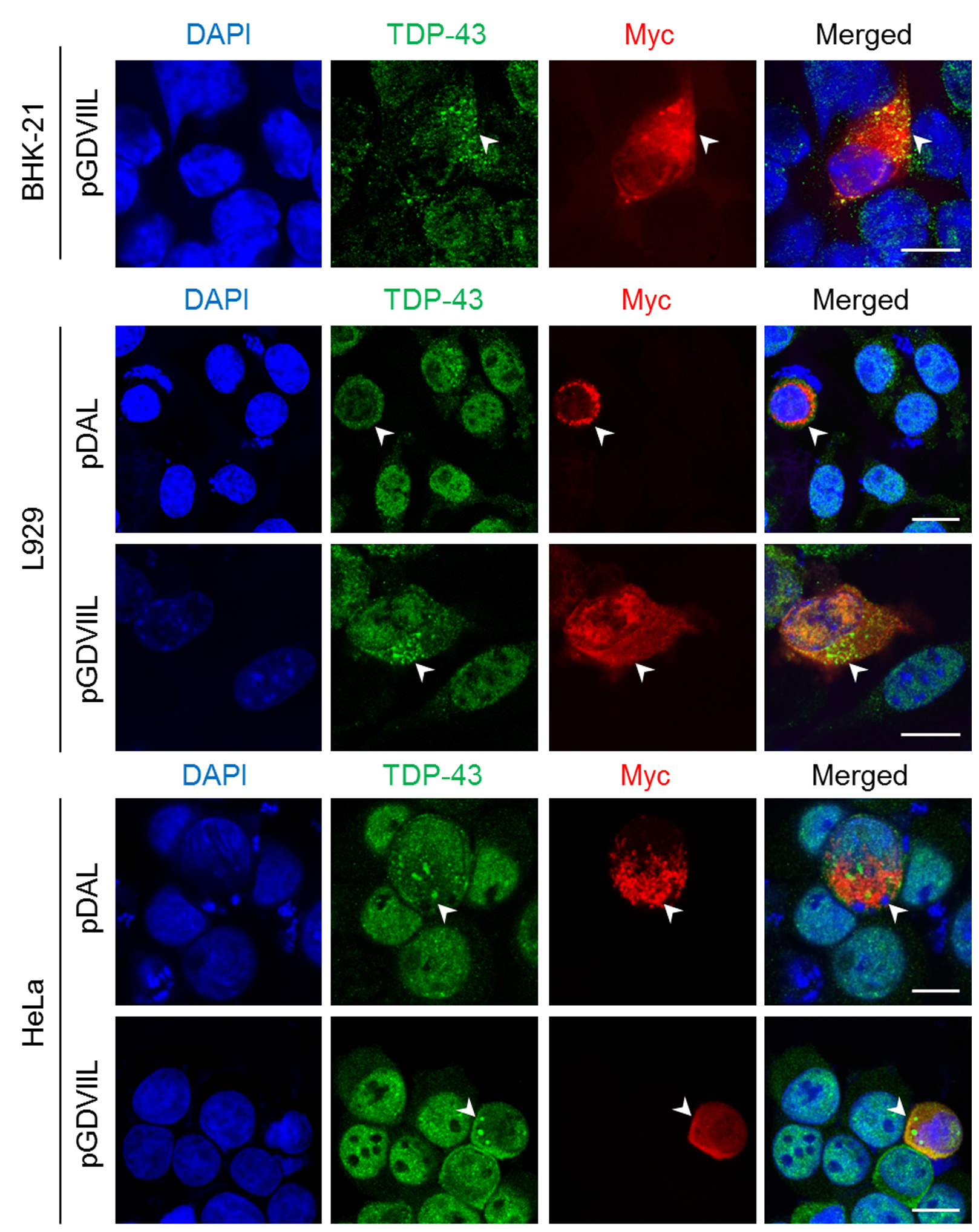

Supplement: S3 Fig — TDP-43 mislocalization and aggregate formation (arrowheads) is present in pDAL- or pGDVIIL-transfected BHK-21, L929, and HeLa cells, The expression of L is indicated by Myc positivity. Scale bars: 10 μm. (TIF) [file ppat.1007574.s003.tif]

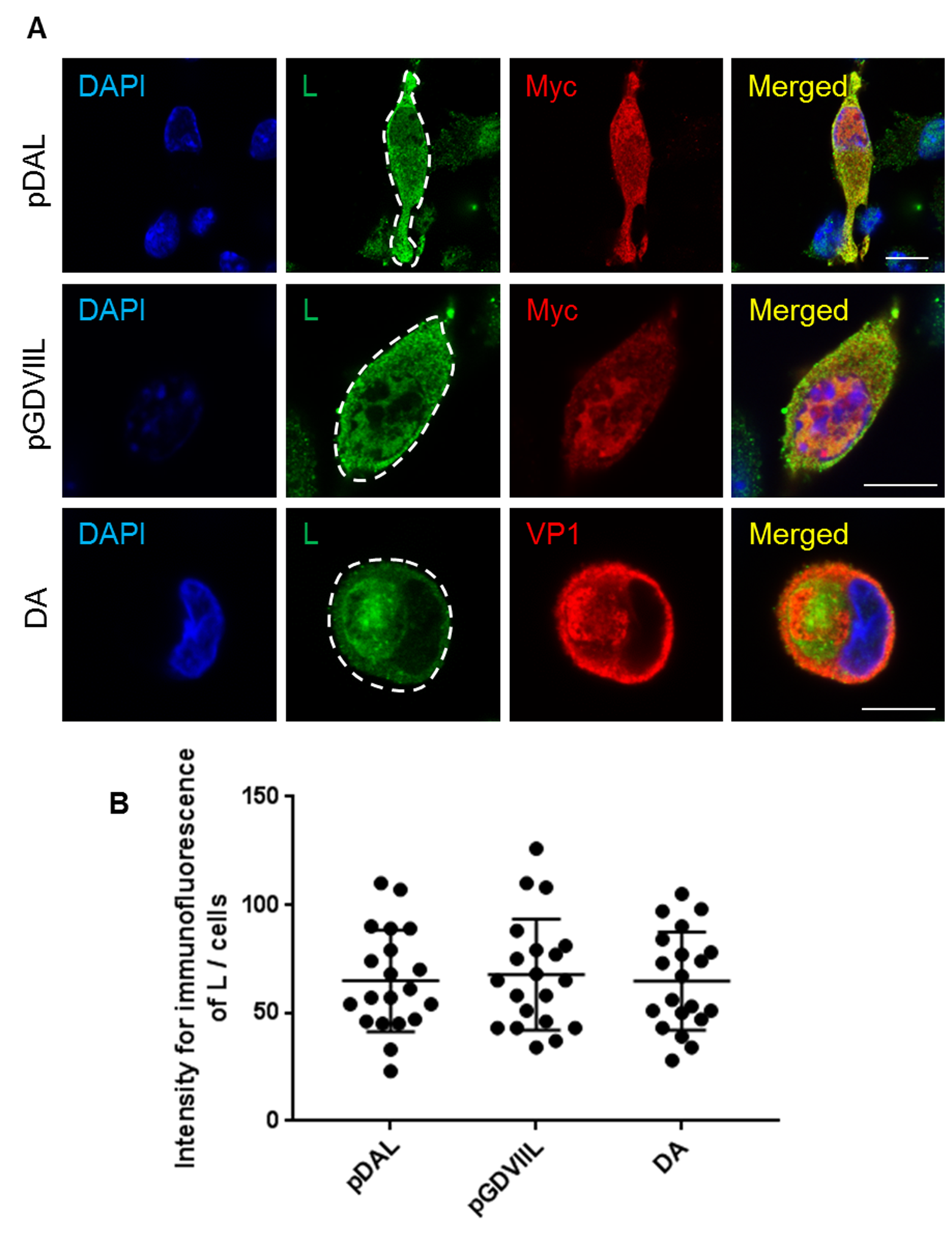

Supplement: S4 Fig — (A) Representative images of immunofluorescence using anti-L antibody. L is expressed in pDAL- or pGDVIIL-transfected BHK-21 cells (that are detected by Myc staining) and DA-infected BHK-21 cells (that are detected by VP1 staining). (B) Intensity of immunofluorescence for L. The Intensity of immunofluorescence of L within each cell (shown, for example, surrounded by a dotted line in (A)) was measured by ImageJ in 20 cells in 5 random fields and was then plotted as a dot graph. The intensity of immunofluorescence in cells from the three groups is not statistically significant. Scale bars: 10 μm. (TIF) [file ppat.1007574.s004.tif]

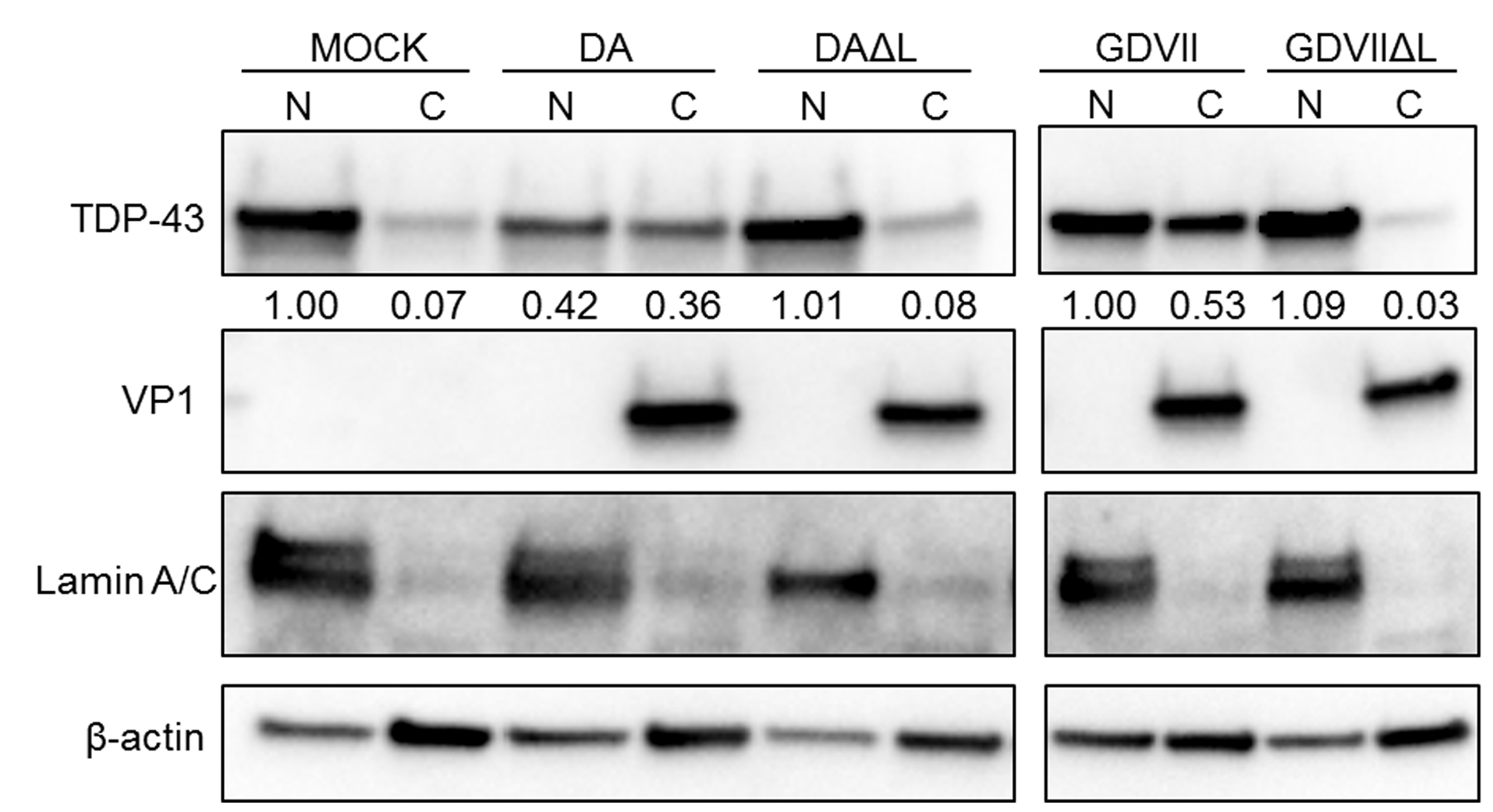

Supplement: S5 Fig — Western blotting of nuclear (N) and cytoplasmic (C) fractions of TMEV-infected BHK-21 cells. The nuclear and cytoplasmic fractions of BHK-21 cells were separated by using NE-PER Nuclear and Cytoplasmic Extraction Reagents (Thermo Fisher Scientific, Waltham, MA). In MOCK, DAΔL and GDVIIΔL-infected cells, TDP-43 is predominantly expressed in the nucleus (and transiently may enter the cytoplasm). In contrast, TDP-43 is significantly mislocalized to the cytoplasm of DA- and GDVII-infected cells. The expression of LaminA/C, a nuclear envelope protein, is primarily in the nuclear fraction, while TMEV VP1 is present in the cytoplasmic fraction. (TIF) [file ppat.1007574.s005.tif]

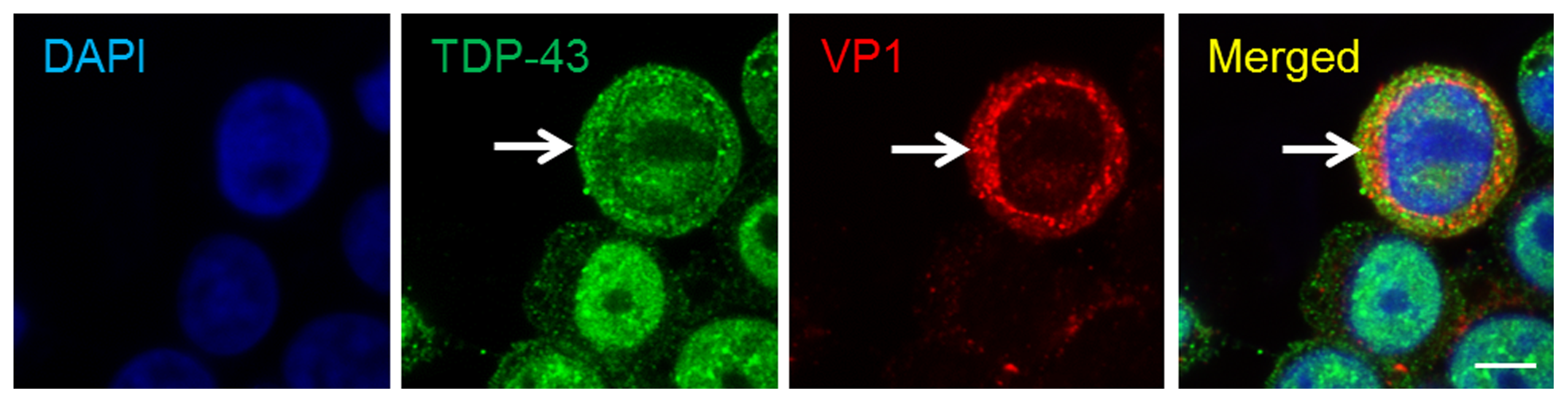

Supplement: S6 Fig — HeLa cells infected by DA virus at 12 HPI. Although TDP-43 is slightly mislocalized to the cytoplasm in VP1-positive cells, aggresomes are not observed in this cell (arrow). Scale bar: 5 μm. (TIF) [file ppat.1007574.s006.tif]

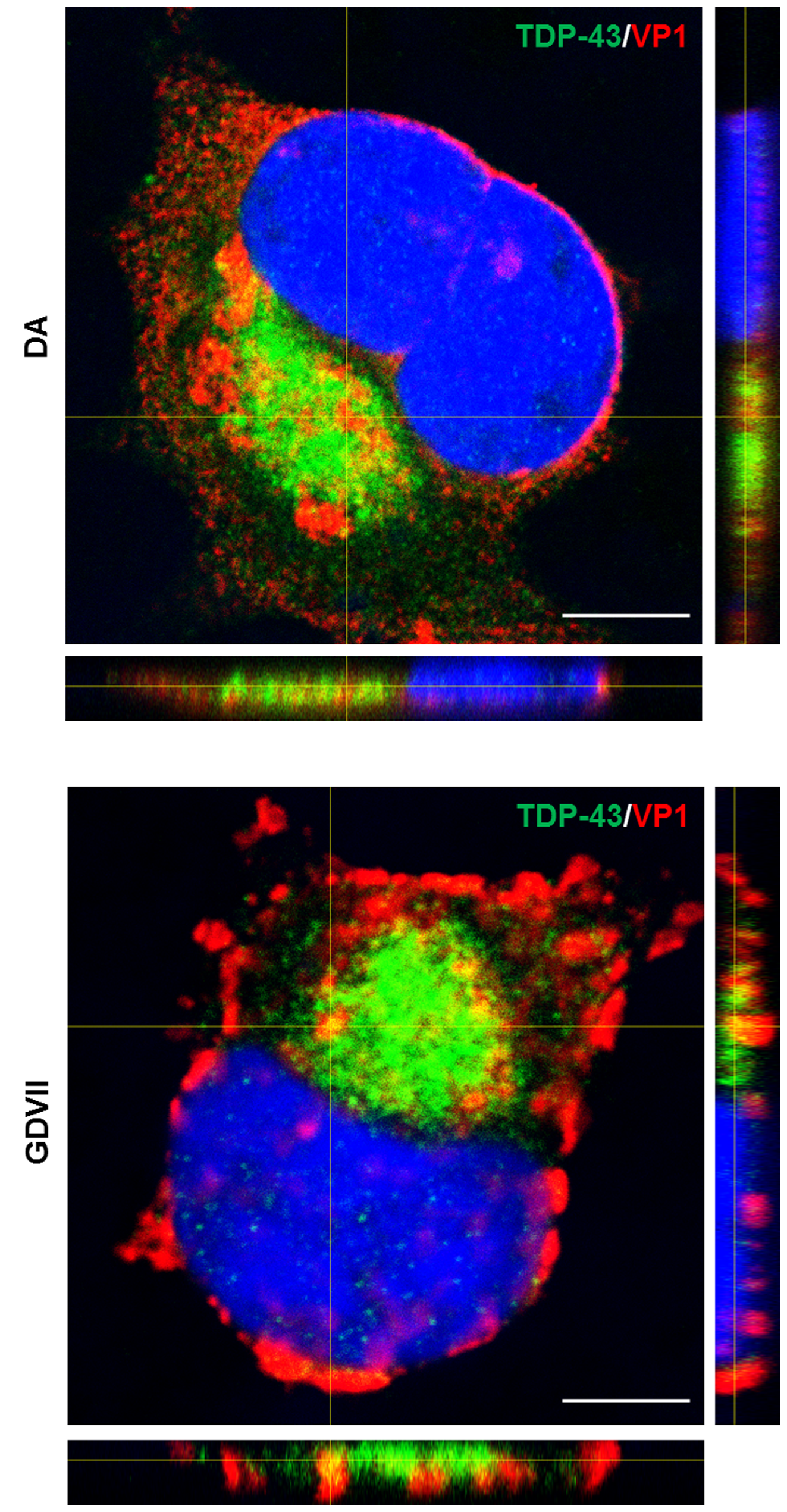

Supplement: S7 Fig — BHK-21 cells infected by DA and GDVII virus at 8 HPI. Both TDP-43 and VP1 accumulate in the juxtanuclear aggresome. TDP-43 and VP1 are partly co-localized within the aggresome shown in yellow. Scale bars: 5 μm. (TIF) [file ppat.1007574.s007.tif]

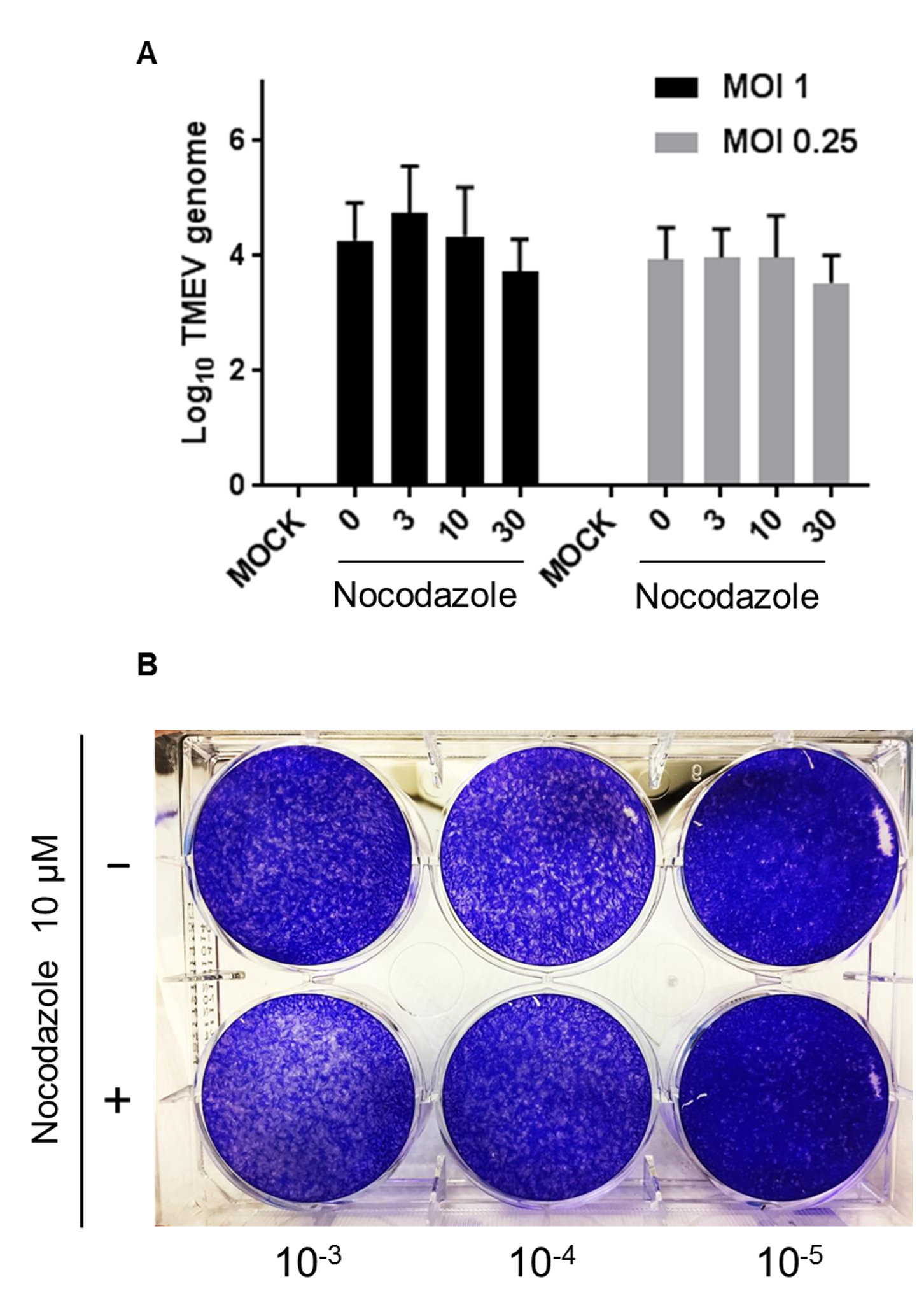

Supplement: S8 Fig — (A) The amount of DA virus genome at 6 HPI with two different MOIs in HeLa cells that had been treated with nocodazole (0, 3, 10, 30 μM) for 1h prior to infection. The viral genome is only slightly decreased with the 30 μM nocodazole treatment. (B) Plaque assay of DA-infected BHK-21 cells treated with nocodazole treatment (10 μM, for 1h prior to infection) shows a similar virus titer compared to untreated cells. (TIF) [file ppat.1007574.s008.tif]

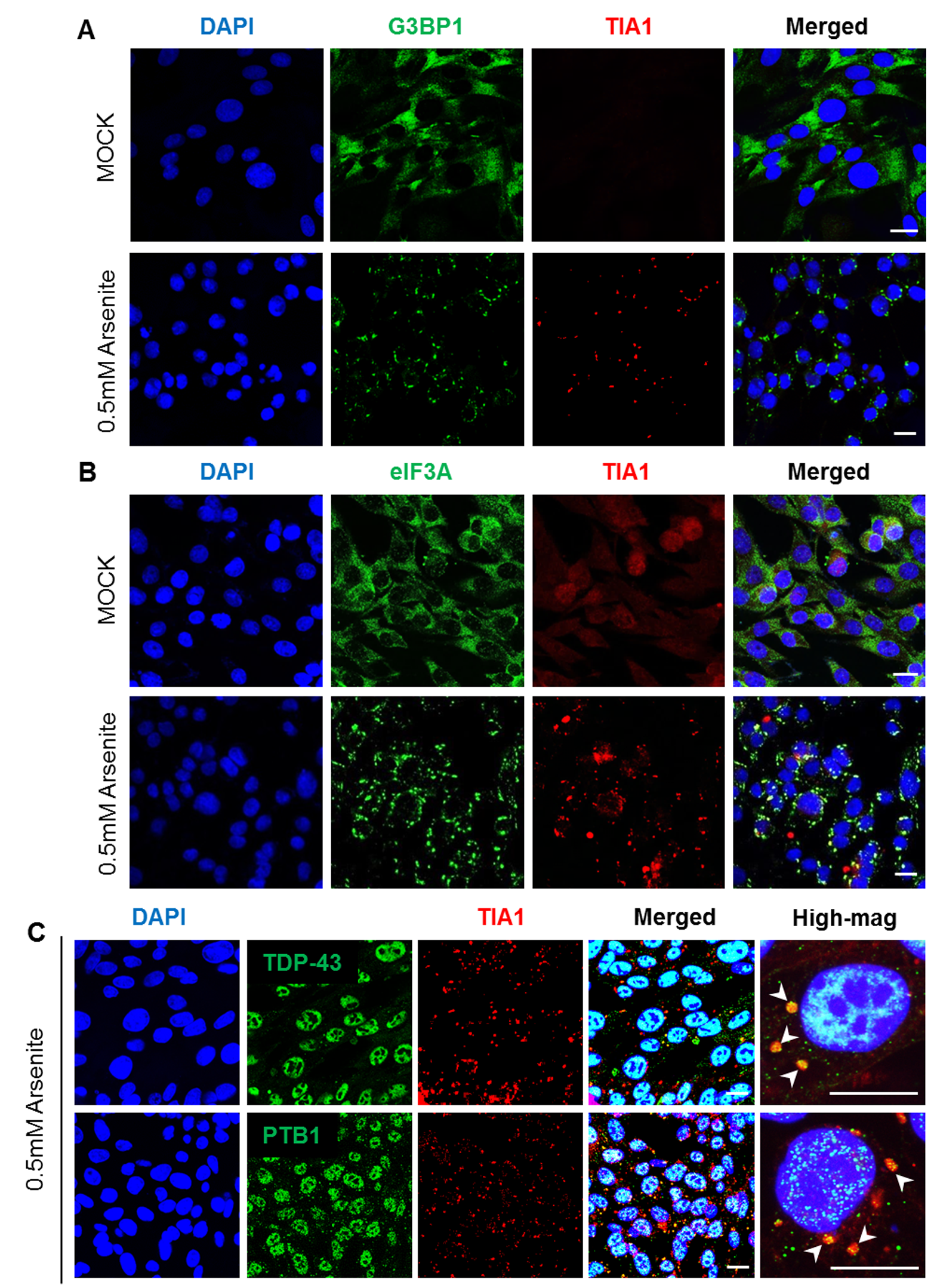

Supplement: S9 Fig — (A-C) BHK-21 cells treated for 45 minutes with 0.5 mM sodium arsenite, a SG inducer, develop SGs that contain SG markers: G3BP1 (A), eIF3A and TIA1 (B). SG markers in mock-treated cells have homogeneous cytoplasmic immunostaining. Following sodium arsenite treatment, SG markers are present in small structures of the typical size of SGs. (C) Following sodium arsenite treatment, TDP-43 and PTB1 partly move into the cytoplasm and merge with TIA1 in SGs (arrowheads). Scale bars: 10 μm. (TIF) [file ppat.1007574.s009.tif]

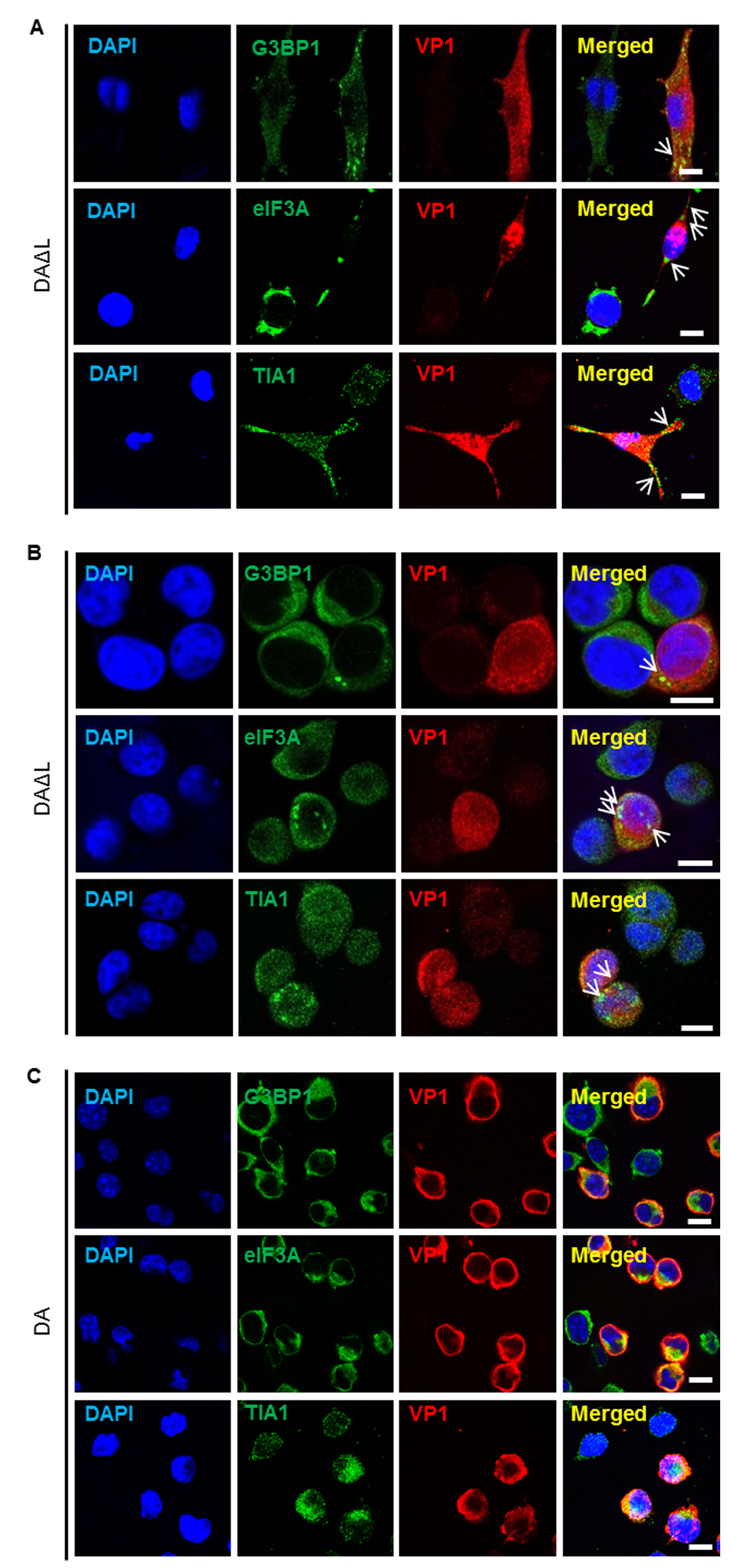

Supplement: S10 Fig — L929 (A) and HeLa (B) cells infected by DAΔL virus at 8 HPI. SGs containing G3BP1, eIF3A and TIA1 are present in the cytoplasm of VP1-positive cells (arrows). (C) L929 cells infected by DA virus at 8 HPI. Aggresomes containing G3BP1, eIF3A and TIA1 are observed in VP1-positive cells. Scale bars: 10 μm (TIF) [file ppat.1007574.s010.tif]

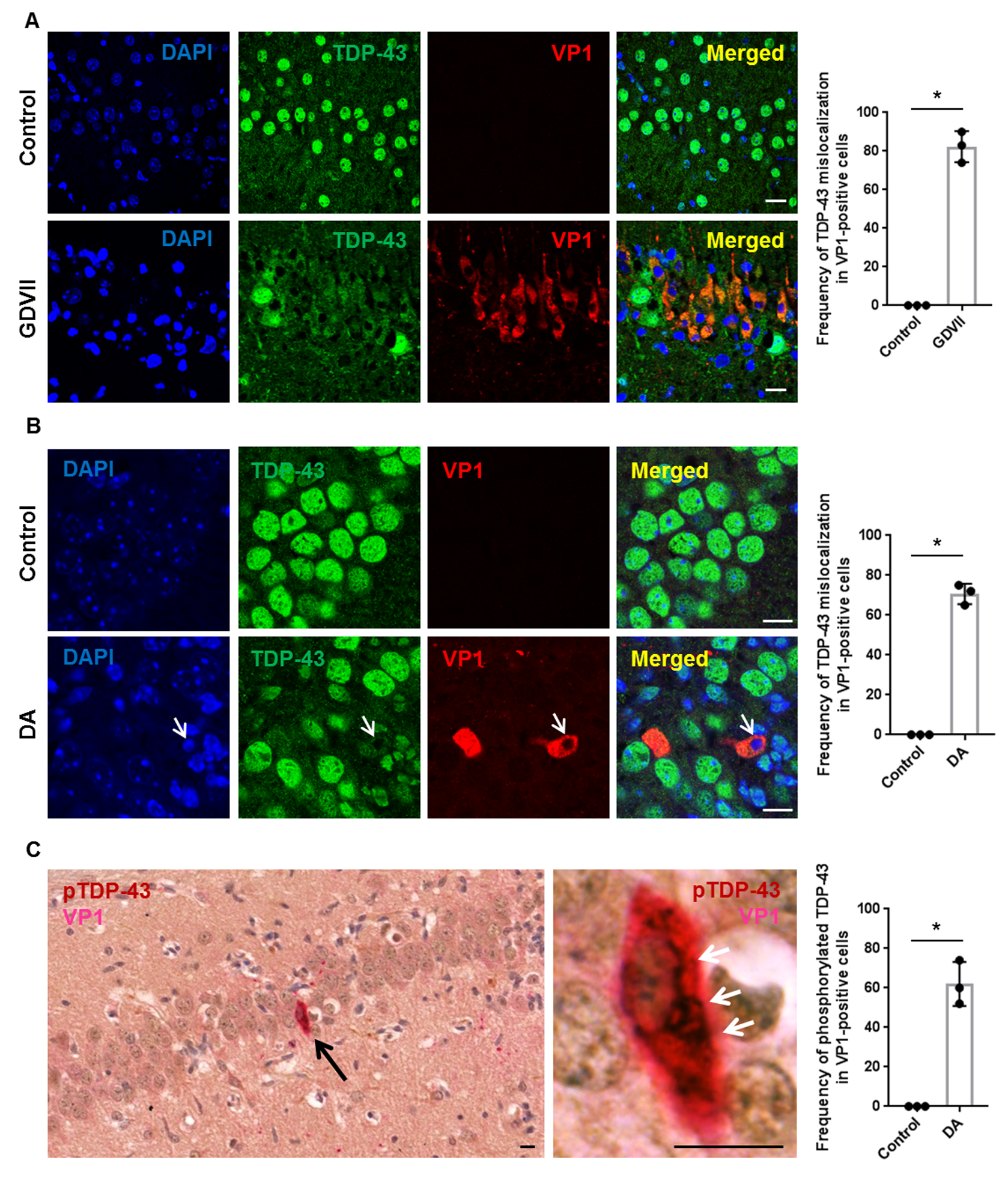

Supplement: S11 Fig — (A, B) Double immunofluorescence for TDP-43 and VP1 in the hippocampus of uninfected and TMEV-infected mice. (A) TDP-43 is predominantly localized to the nucleus of CA1 region neurons in uninfected mice. In contrast, TDP-43 is depleted in the nucleus, and mislocalized to the cytoplasm of VP1-positive neurons in CA1 region 1 week after infection with GDVII virus. The frequency of TDP-43 mislocalization in VP1-positive cells is ~80%, as shown in the graph bar (n = 3). (B) TDP-43 is depleted in the nucleus and mislocalized to the cytoplasm in VP1-positive neurons (arrows) in CA2 region two weeks after infection of DA virus. The frequency of TDP-43 mislocalization in VP1-positive cells is ~70%, as shown in the graph bar (n = 3). (C) Representative image showing phosphorylation of TDP-43 in VP1-positive CA1 region neuron (arrow) two weeks after infection of DA virus. Higher magnification shows skein-like inclusion (arrows) which is immunopositive for pTDP-43 (brown) in a VP1-positive cell (pink). The frequency of TDP-43 phosphorylation in VP1-positive cells is ~60%, as shown in the graph bar (n = 3). Scale bars: 10 μm. *P < 0.0001. (TIF) [file ppat.1007574.s011.tif]
