# Supplementary material for: TDP-43 proteinopathy in Theiler’s murine encephalomyelitis virus infection
Source: PLoS Pathog. 2019 Feb 11;15(2):e1007574. doi: 10.1371/journal.ppat.1007574 (PMC6390522; doi:10.1371/journal.ppat.1007574)
Supplement: S1 Table — (DOCX) [file ppat.1007574.s012.docx]

# **S1 Table** Antibodies used for immunocytochemistry

| **Antigen** |  | **Type** | **Dilution** | **Source** |
| --- | --- | --- | --- | --- |
| RNA-binding protein | TDP-43 (N-terminal) | Rabbit polyclonal | 1:200 | Proteintech, Rosemont, IL, USA |
|  | TDP-43 (1D3, phospho-S409/410) | Rat monoclonal | 1:100 | EMD Millipore, Burlington, MA, USA |
|  | TDP-43 (phospho-S409/410) | Rabbit polyclonal | 1:100 | Proteintech, Rosemont, IL, USA |
|  | PTB1 | Rabbit polyclonal | 1:200 | Abcam, Cambridge, UK |
|  | FUS | Rabbit polyclonal | 1:200 | Proteintech, Rosemont, IL, USA |
| Stress granule marker | G3BP1 | Rabbit polyclonal | 1:100 | Abcam, Cambridge, UK |
|  | eIF3A | Rabbit polyclonal | 1:200 | Abcam, Cambridge, UK |
|  | TIA1 | Rabbit polyclonal | 1:200 | Proteintech, Rosemont, IL, USA |
| Cytoskeleton protein | Vimentin | Rabbit polyclonal | 1:100 | Invitrogen, Carlsbad, CA, USA |
| Viral component | VP1 | Mouse monoclonal | 1:300 | Nitayaphan, S, 1985 |
|  | L | Rabbit polyclonal | 1:100 | Purified IgG from sera of rabbits immunized against recombinant L protein |
|  | L* | Rabbit polyclonal | 1:100 | Gift from Dr. Yoshiro Ohara, Japan |
|  | ds-RNA (K1) | Mouse monoclonal | 1:200 | Scicons, Szirák, Hungary |
| Tag | Myc | Rabbit polyclonal | 1:1000 | Cell Signaling Technology, Danvers, MA |
