# Supplementary material for: TDP-43 proteinopathy in Theiler’s murine encephalomyelitis virus infection
Source: PLoS Pathog. 2019 Feb 11;15(2):e1007574. doi: 10.1371/journal.ppat.1007574 (PMC6390522; doi:10.1371/journal.ppat.1007574)
Supplement: S2 Table — (DOCX) [file ppat.1007574.s013.docx]

# **S2 Table** Antibodies used for immunohistochemistry

| **Antigen** |  | **Type** | **Dilution** | **Source** |
| --- | --- | --- | --- | --- |
| RNA-binding protein | TDP-43 (N-terminal) | Rabbit polyclonal | 1:200 | Proteintech, Rosemont, IL, USA |
|  | TDP-43 (1D3, phospho-S409/410) | Rat monoclonal | 1:100 | EMD Millipore, Burlington, MA, USA |
|  | PTB2 | Rabbit polyclonal | 1:200 | Abcam, Cambridge, UK |
| Viral component | VP1 | Mouse monoclonal | 1:300 | Nitayaphan, S, 1985 |
| Oligodendrocyte/myelin | CNPase | Rabbit monoclonal | 1:1000 | Cell Signaling Technology, Danvers, MA |
|  | CNPase | Mouse monoclonal | 1:400 | Abcam, Cambridge, UK |
| Microglia/macrophage | Iba-1 | Rabbit polyclonal | 1:1000 | WAKO, Osaka, Japan |
